# Dual-sensitive antibacterial peptide nanoparticles prevent dental caries

**DOI:** 10.7150/thno.73181

**Published:** 2022-06-13

**Authors:** Peng Zhang, Saizhi Wu, Jinting Li, Xiaoshuang Bu, Xiaoping Dong, Ninglin Chen, Fengjiao Li, Jingyu Zhu, Longkang Sang, Youlin Zeng, Songping Liang, Zhilin Yu, Zhonghua Liu

**Affiliations:** 1 The National and Local Joint Engineering Laboratory of Animal Peptide Drug Development, College of Life Sciences, Hunan Normal University, Changsha, Hunan, China.; 2 Key Laboratory of Functional Polymer Materials, Ministry of Education, College of Chemistry, Nankai University, Tianjin, Tianjin, China.; 3 Department of Periodontology and Pediatric Dentistry, Changsha Stomatological Hospital, School of Dental Medicine, Hunan University of Chinese Medicine Changsha, Hunan, China.; 4 The National and Local Joint Engineering Laboratory for New Petrochemical Materials and Fine Utilization of Resources, College of Chemistry and Chemical Engineering, Hunan Normal University, Changsha, Hunan, China.

**Keywords:** antimicrobial peptides, stimuli-responsive, conformational transition, nanoparticles, biofilms, dental caries

## Abstract

**Background:** Dental caries is the most prevalent bacterial biofilm-induced disease. Current clinical prevention and treatment agents often suffer from adverse effects on oral microbiota diversity and normal tissues, predominately arising from the poor biofilm-targeting property of the agents.

**Methods:** To address this concern, we herein report dual-sensitive antibacterial peptide nanoparticles pHly-1 NPs upon acid and lipid-binding for treatment of dental caries. Amino acid substitutions were performed to design the peptide pHly-1. The potential, morphology and secondary structure of pHly-1 were characterized to elucidate the mechanisms of its pH and lipid sensitivity. Bacterial membrane integrity assay and RNA-seq were applied to uncover the antimicrobial mechanism of peptides under acidic condition. The *in vitro* and *ex vivo* antibiofilm assays were used to determine the antibiofilm performance of pHly-1 NPs. We also carried out the *in vivo* anti-caries treatment by pHly-1 NPs on dental caries animal model. Oral microbiome and histopathological analyses were performed to assess the *in vivo* safety of pHly-1 NPs.

**Results:** The pHly-1 peptide underwent the coil-helix conformational transition upon binding to bacterial membranes in the acidic cariogenic biofilm microenvironment, thereby killing cariogenic bacteria. Under normal physiological conditions, pHly-1 adopted a *β*-sheet conformation and formed nanofibers, resulting in negligible cytotoxicity towards oral microbes. However, in acidic solution, pHly-1 NPs displayed reliable antibacterial activity against *Streptococcus mutans*, including standard and clinically isolated strains, mainly via cell membrane disruption, and also suppressed *in vitro* and human-derived *ex vivo* biofilm development. Compared to the clinical agent chlorhexidine, *in vivo* topical treatment with pHly-1 NPs showed an advanced effect on inhibiting rat dental caries development without adverse effects on oral microbiota diversity and normal oral or gastric tissues.

**Conclusion:** Our results demonstrated the high efficacy of dual-sensitive antimicrobial peptides for the selective damage of bacterial biofilms, providing an efficient strategy for preventing and treating dental caries.

## Introduction

Dental caries (tooth decay) is the most prevalent oral disease, which seriously endangers human public health and is costly, with an estimated cost over $120 billion per year in the USA alone 1,2. Previous studies have demonstrated that dental caries are driven by dysbiosis of microbial biofilm under sugar-rich and acidic conditions with subsequent demineralization of tooth tissues 3. Animal experiments and epidemiological and clinical studies have shown that dental caries are associated with *Streptococcus mutans* (*S. mutans*), which plays an important role in microbial biofilm development, especially for early childhood caries 3-5. Killing cariogenic bacteria and inhibiting biofilm development are critical to preventing and treating dental caries. Many antimicrobial agents (e.g., chlorhexidine, CHX) and naturally occurring antibiofilm drugs (e.g., terpenoids) can effectively treat dental caries 6. However, they suffer from adverse effects due to the limited selectivity against cariogenic bacteria and biofilms 7, poor aqueous solubility, and among others 6,8. Although antibiotics eliminate superficial pathogenic bacteria and prevent dental caries development, they lead to drug resistance and normal indigenous microflora disruption 6,9-11. Therefore, the development of antibacterial agents for efficiently damaging cariogenic bacterial biofilms is particularly urgent and significant for the prevention and treatment of dental caries.

In the presence of dietary sugars, integration of the extracellular polymeric substances matrices with the embedded acidogenic oral pathogens *S. mutans* creates protective and acidic cariogenic biofilms on tooth surfaces with pH values ranging from 4.5 to 5.5 4,12. However, oral commensal bacteria/normal tissues often exhibit relatively neutral pH values (6.8-7.0). The different pH features of the cariogenic biofilms and normal tissues led to the development of various nanomaterials with selective anti-biofilm activity towards acidic cariogenic biofilms for dental caries management. Koo et al. reported a series of biofilm microenvironment-responsive (e.g., pH) nanoparticles such as Dex-NZM 13, Farnesol-NPC 14, CAT-NP/H_2_O_2_ 15, and ferumoxytol/H_2_O_2_ 12 for targeted therapy against dental caries without adverse effects on oral microbiota diversity and mucosal and gingival tissues. Also, acid-activated antimicrobial peptides have been developed based on their broad-spectrum antibacterial property and low bacterial resistance. For instance, Cheng and coauthors reported polypeptides undergoing the pH-responsive helix-coil conformation transition and killing *H. pylori* in the stomach 16. Despite their great clinical application prospect, precise activation of the antibacterial agents remains challenging, indicating the demand for multi-responsive antibacterial agents for selective treatment of dental caries.

Peptides are a promising class of molecules exhibiting broad bioactivity and biocompatibility 17. In particular, antimicrobial peptides (AMPs) are promising candidates 18 that mainly disrupt bacterial membrane structure. In addition, AMPs exhibit strong activity in inhibiting pathogenic biofilm formation, which is important in treating biofilm-induced diseases 18-21. The antibacterial activity of peptides is conventionally associated with their conformation that could be manipulated by different stimuli, such as pH, light, enzymes, and the microenvironment 22,23. For example, AMP PHOPT-2 could be specifically activated by bacterial phosphatase at the infectious site via random coil-to-helix transition and exhibited low toxicity against mammalian cells 24. These considerations motivated us to develop dual-sensitive antibacterial peptide nanoparticles for selective and efficient treatment of dental caries.

**Scheme 1:**
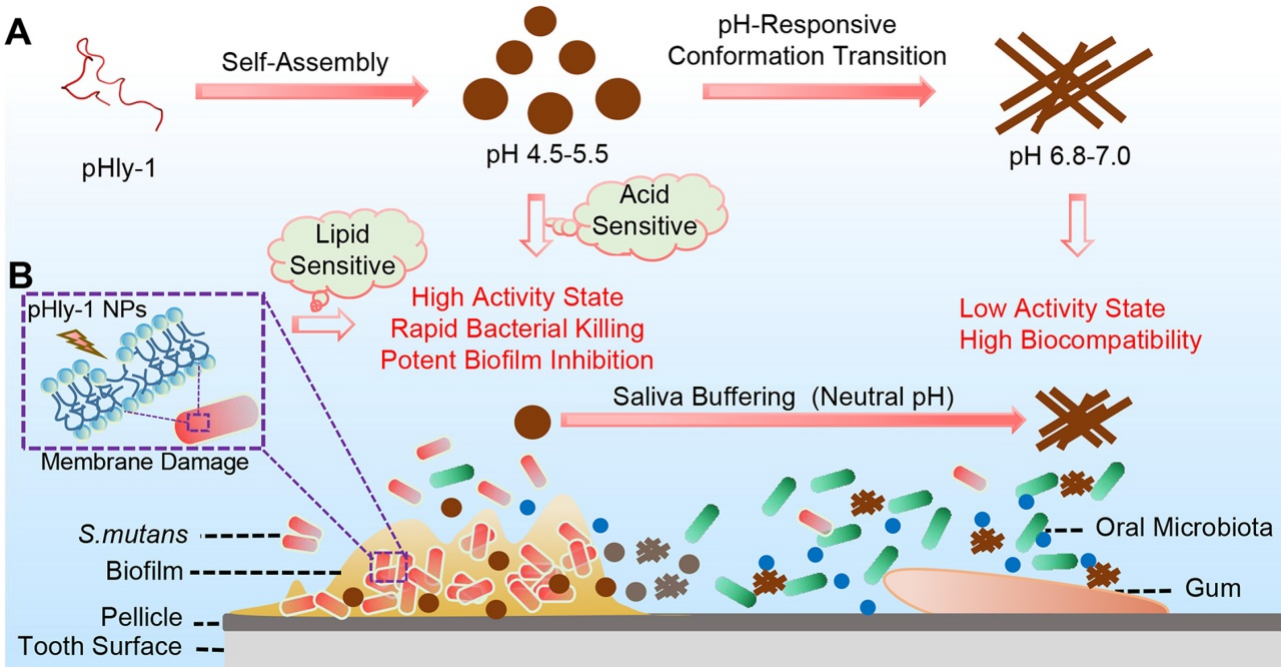
** (A)** Illustration of the pH-responsive self-assembly and morphological transition of peptide pHly-1 from nanoparticles to nanofibers upon increasing solution pH. **(B)** The process of treating dental caries by peptide pHly-1, in which pHly-1 NPs bind to the bacterial membranes under acidic cariogenic biofilm microenvironment and thereby promoting the coil-helix conformational transition and penetrating of the peptide into membranes for killing bacteria. The diffusion of the peptide in the neutral saliva leads to the conformational transition into β-sheets and formation of nanofibers, thus limiting the interaction between pHly-1 with bacterial membranes and the low cytotoxicity to oral microbes and gingival and mucosal tissues.

Herein we report on an antibacterial peptide with activity sensitive to the acid condition and the lipid-binding ability for the treatment of dental caries (Scheme 1). The dual-sensitive antimicrobial peptide pHly-1 was designed by primarily replacing lysine with histidine residues in lycosin-I, a 24-residue cationic linear peptide isolated from the venom of the spider* Lycosa singorensis,* with broad-spectrum antibacterial activity 25,26, Peptide pHly-1 adopts random coil conformation under acidic conditions and forms nanoparticles (NPs). In contrast, exposure to neutral pH leads to a conformational transition to form *β*-sheets and nanofibers. Under the acidic condition in the cariogenic biofilm microenvironment, binding with the bacterial membrane promotes the coil-helix conformation transition of peptide pHly-1, thereby penetrating the lipid bilayer and damaging *S. mutans* biofilm. However, the *β*-sheet pHly-1 nanofibers at neutral pH maintain aggregated state, stabilized by hydrogen bonding and hydrophobic interactions, limiting the penetration of pHly-1 into bacterial membranes and resulting in negligible toxicity to oral microbes and gingival and mucosal tissues. In this study, we thoroughly investigated the performance of pHly-1 NPs in inhibiting the *in vitro* and* ex vivo* biofilm development and evaluated its effect on the *in vivo* topical treatment of dental caries.

## Results

### Peptide pHly-1 undergoes pH- and lipid-responsive conformational transition

**Figure 1:**
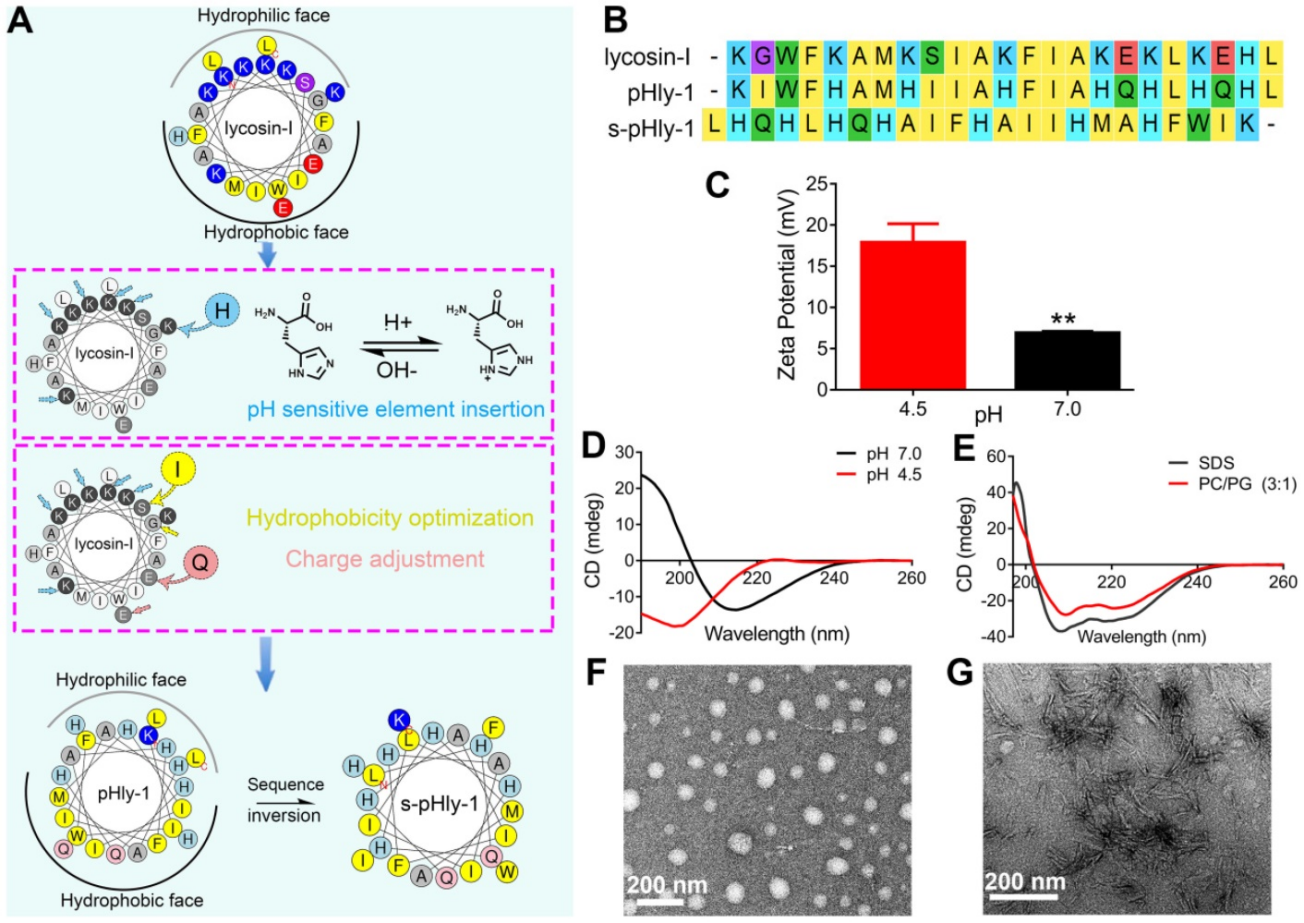
** Designed of peptide pHly-1 and its pH- or lipid-responsive conformation. (A)** Design process of the sequence of pHly-1 by amino acids substitution. The pH-sensitive AMP pHly-1 was derived from the sequence of lycosin-I by lysine replacement, histidine addition, charge adjustment and hydrophobicity optimization. (**B**) The sequences of lycosin-I, pHly-1 and s-pHly-1 were aligned by using MEGA soft. (**C**) The ζ-potential of pHly-1 (100 µM) at pH 4.5 and 7.0. Unpaired two-tailed Student's t-test was used for statistical analysis. Data are shown as mean ± s.d.; n = 4. (**D**) CD spectra of peptide pHly-1 (100 µM) in aqueous solution at pH 4.5 and 7.0. (**E**) CD spectra of peptide pHly-1 (100 µM) in 100 mM SDS or PC/PG (3:1) at pH 4.5. (**F** and **G**) TEM images of the assemblies of pHly-1 at pH 4.5 (**F**) and 7.0 (**G**).

As shown in Figure 1A, pHly-1 was designed via amino acid substitution using lycosin-I as a template involving multiple steps. (1) Considering the importance of the positive charge in pH-responses of peptides, histidine was used to selectively replace the lysine residues in lycosin-I as the pH-responsive moiety to control the assembly and disassembly of a peptide due to its relatively low pKa value compared to lysine. (2) The electrostatic interaction between cationic AMPs and microbial membranes with negative charges of lipids, such as phosphatidylglycerol (PG), is the initial step in exerting antibacterial activity 27-29. Hence, the negatively charged glutamic acid residues within lycosin-I were exchanged for neutral glutamine residues. (3) Hydrophobicity is essential for the membrane disruption activity of antimicrobial peptides for their interaction and partitioning into the membrane layers 27,28,30,31. To enhance the antimicrobial activity of lycosin-I at acidic pH and increase the interaction between peptides under neutral conditions, hydrophobicity of peptides was optimized by substituting less hydrophobic amino acids glycine and serine with isoleucine. Simultaneously, a reserve peptide s-pHly-1 was designed and synthesized as the reference peptide by maintaining the residue constituents. All peptide variants were successfully obtained by Fmoc-solid-phase synthesis. Finally, a variant peptide pHly-1 was obtained (Figure 1A-B). The helical wheel structures of the peptides were predicted by heliquest, showing the separated hydrophobic and hydrophilic faces of the resulting peptide when adopting an α-helical conformation (Figure 1A). Theoretically, compared with lycosin-I, the calculated hydrophobicity value of pHly-1 was increased from 0.299 to 0.786 and the hydrophobic face was also increased accordingly (Figure S1).

To confirm the potential acid- and lipid-responsive performance of pHly-1, we measured a series of physicochemical parameters at different conditions. The ζ-potential of pHly-1 at pH 4.5 and pH 7.0 was estimated to be 17.9 ± 2.25 mV and 6.9 ± 0.18 mV, respectively (Figure 1C). This might be attributed to the protonation/deprotonation of the histidine residues in the pHly-1 sequence in response to pH changes when most histidine residues were neutral at pH 7.0, while they were protonated and positively charged at pH 4.5. The secondary structure of pHly-1 peptide at pH 4.5 and pH 7.0 was investigated by circular dichroism (CD) spectroscopy. At pH 7.0, the CD spectrum of pHly-1 displayed a positive band at 195 nm and a negative one at 216 nm in an aqueous solution (Figure 1D), indicating the formation of *β*-sheets by pHly-1. In contrast, at pH 4.5, a negative band at 200nm was observed in the CD spectrum of pHly-1, suggesting its random coil conformation (Figure 1D). In the presence of lipid vesicles formed by POPC/POPG, employed as model membranes, pHly-1 showed the typical helical conformation as evidenced by the double minima at 208 and 222 nm (Figure 1E). Furthermore, pHly-1 also displayed the helical conformation at pH 4.5 and 7.0 in the 100 mM SDS buffer due to the formed hydrophobic environment analogous to cell membranes (Figure 1E and Figure S2A). These results demonstrated the conformational transition for peptide pHly-1 between random coils and *β*-sheets as well as random coils and helices promoted by acid change and lipid bilayer-binding, respectively. Morphological studies by transmission electron microscopy (TEM) demonstrated that pHly-1 formed nanoparticles with an average diameter of approximately 40 nm at pH 4.5 (Figure 1F), whereas the length of nanofibers formed by the peptide was about 400 nm at pH 7.0 (Figure 1G). These data were also confirmed by AFM experiments (Figure S2B).

### pHly-1 displays pH- and lipid binding-sensitive antimicrobial activity

**Figure 2:**
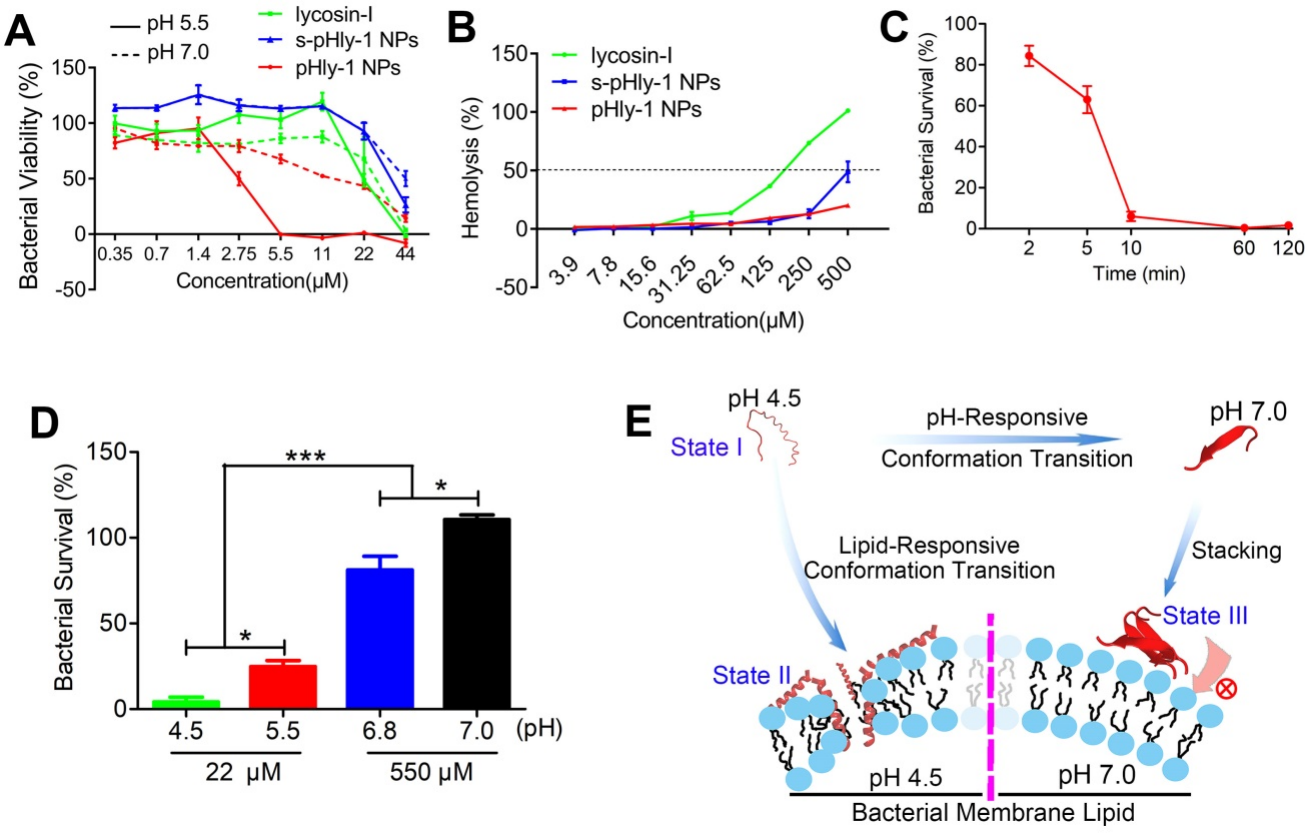
** Characterizations of the antimicrobial activity of peptide pHly-1. (A)** The MIC values of peptides lycosin-I, pHly-1, and s-pHly-1 against *S. mutants* UA159. The bacteria were incubated in the presence of peptides with various concentrations at pH 5.5 or 7.0 for 48 h. The bacterial viability(%) = [(A_600_ sample-A_600_ blank)/(A_600_ negative control-A_600_ blank)] × 100%. **(B)** The hemolytic activity of peptides lycosin-I, pHly-1, and s-pHly-1. Lycosin-I, pHly-1 and s-pHly-1 were dissolved in PBS (pH 7.4) at various concentrations, and incubated with fresh mice erythrocytes for 30 min. **(C)** The bacteria-killing kinetic curve for pHly-1 against *S. mutans* UA159. The bacteria were treated with pHly-1 at a concentration of 22 µM at pH 4.5. **(D)** The bacterial killing activity of pHly-1 against *S. mutans* UA159. The bacteria were treated with 22 (4 MIC) or 550 µM (100 MIC) of pHly-1 for 10 min under acidic conditions (4.5 and 5.5) and neutral conditions (6.8 and 7.0), respectively. Bacterial survival (%) = CFU of bacteria treated with peptides/CFU of bacteria treated with negative control × 100%. All results are presented as mean ± s.d. (n = 3).** (E)** Schematic illustration of the pH- and lipid-responsive conformational transition of peptide pHly-1 and its antibacterial activity associated with the states sensitive to the acidic condition and lipid bilayer penetration.

The antimicrobial activity of lycosin-I peptides, pHly-1 and s-pHly-1 was evaluated via measuring with their minimum inhibitory concentrations (MICs) against *S. mutans* UA159 at acidic and neutral pH values, while their toxicity was determined by the hemolysis assay. Lycosin-I possesses rapid, potent, and broad-spectrum antimicrobial activity to clinically isolated multidrug-resistant *Acinetobacter baumannii* (MDRAB) 25 and 27 strains of microbes 26. However, the MIC value of lycosin-I against *S. mutans* UA159 was approximately 44 μM at both pH 5.5 and 7.0 (Figure 2A and Table S1), indicating the pH-independent weak antibacterial effect of lycosin-I on *S. mutans*. The MIC value of pHly-1 NPs against *S. mutans* UA159 was 5.5 μM and >44 μM at pH 5.5 and 7.0, respectively. It is of note that investigating the antimicrobial activity of pHly-1 at pH 7.0 is to mimic the mild acidic microenvironment of *S. mutans* UA159 which usually releases proton to culturing media and thereby decreasing the medium pH (Figure S3A). To rule out the contribution of the acid type to the antibacterial activity, the MIC study was performed when the acidic BHI medium was tuned by different acid solutions. As shown in Figure S3B, MIC values of pHly-1 NPs were constant across different types of acid solutions, thus excluding the acid effect on the antibacterial activity. Importantly, treatment of red blood cells for 30 min with 500 μM pHly-1 NPs only led to about 20% hemolysis. However, 180 μM lycosin-I resulted in ~50% hemolysis (Figure 2B and Table S1) and S-pHly-1 with the reversed sequence of pHly-1 exhibited much lower antimicrobial activity (Figure 2A and Table S1), indicating that the critical role of the amino acid sequence of pHly-1 in its activity. These results showed pHly-1 as a pH-responsive AMP capable of killing acidogenic oral pathogen *S. mutans* under the acidic condition.

**Figure 3:**
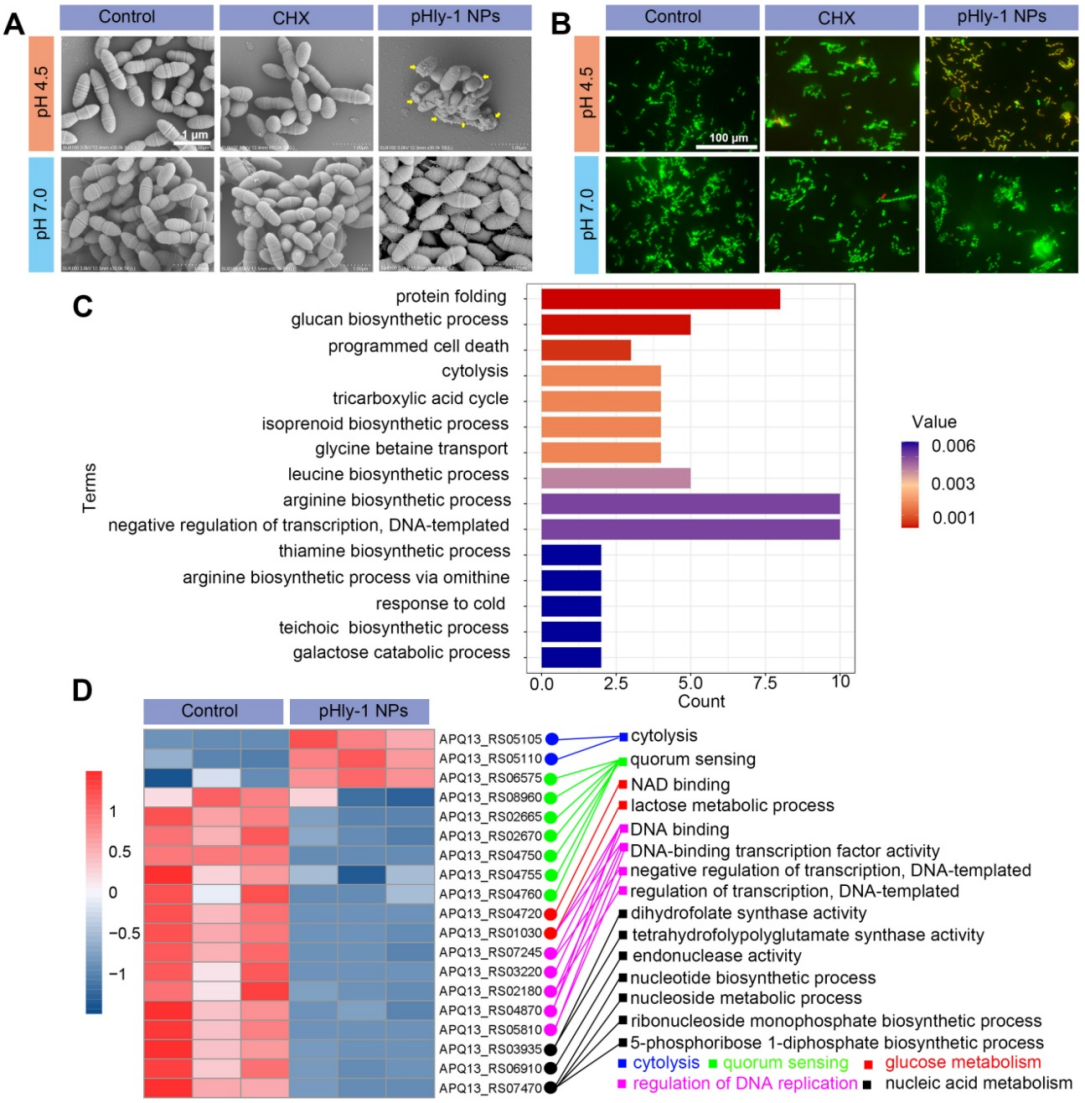
** Mechanistic studies for antimicrobial activity of pHly-1 NPs. *S. mutans* UA159 was treated with 22 μM pHly-1 NPs or CHX at different pH for 1 h and then submitted for microscopic studies, fluorescent staining and transcriptomic analyses. (A)** SEM images of *S. mutans* after treatment with PBS aqueous solution (control), pHly-1 NPs or CHX at pH 4.5 (top) and pH 7.0 (bottom), respectively. Yellow arrows represent fragmented morphology of *S. mutans* cells. Scale bar, 1 m. **(B)** Dual staining images of *S. mutans* after treatment with aqueous solution, pHly-1 NPs and CHX at pH 4.5 (top) and pH 7.0 (bottom) for 1 h. Living and dead *S. mutans* UA159 cells were labeled in green by SYTO 9 or in red by propidium iodide (PI), respectively. Scale bar, 100 µm. **(C)** Gene functional annotation enrichment analysis of the DEGs of *S. mutans* obtained from transcriptome analysis. **(D)** Cluster heat map of the expression differences of some *S. mutans* genes regulating the bacterial survival-related processes including cytolysis, quorum sensing, glucose metabolism, DNA replication regulation and nucleic acid metabolism.

CHX, considered as the 'gold standard' for oral antimicrobial therapy 32,33, was used as the reference sample to decipher the pH-sensitive antibacterial activity of pHyl-1. The MIC values of CHX against *S. mutans* UA159 were estimated to be 0.5 and 1 μM at pH 5.5 and 7.0, respectively (Figure S3C). Despite the stronger activity compared to pHly-1 NPs, the antimicrobial activity of CHX is almost pH-independent. Then, the bacterial killing assay was performed and quantitative analysis was determined by total viable cell counting (colony forming units, CFU). The bacteria death kinetic assay showed that the treatment with 22 μM pHly-1 NPs for 10 min resulted in almost complete bacterial death, whereas ca. about 60% bacterial survival remained upon treatment with the same concentration of CHX for 120 min (Figure 2C and Figure S3D). We further compared their rapid bacterial killing activities determined by the bactericidal ability against *S. mutans* UA159 following treatment for 10 min. As shown in Figure 2D and Figure S3E, 22 μM pHly-1 NPs killed ~60% and ~95% *S. mutans* UA159 at pH 5.5 and 4.5, respectively, in contrast to the negligible bactericidal effect at pH 6.8 and 7.0 even at 550 μM. These results confirmed the pH-dependent bactericidal activity of pHly-1 NPs, and therefore it is reasonable for the improved bacterial death induced by pHly-1 NPs in acidic media. In the case of CHX, no apparent bactericidal effect at 22 μM was observed at both acidic and neutral pH; however, 550 μM CHX could kill bacteria completely under both acidic and neutral conditions (Figure 3SF). The bactericidal ability under the four pH conditions was also confirmed the best antibacterial activity of pHly-1 NPs at pH 4.5 (Figure S3G). These data indicate the potent and rapid bacterial killing activity of pHly-1 NPs under acidic conditions.

Based on the lipid-responsive conformational and morphological transition of pHly-1 and its pH-dependent antibacterial activity (Figure 1), we hypothesized the underlying mechanism for its antimicrobial activity, depicted in Figure 2E. The protonation of histidine residues under the acidic condition is the key factor in determining pHly-1 conformations (Figure S4). Below pH 6.0, the imidazole groups of the seven histidine residues (pKa ≈ 6.0) of pHly-1 are protonated, leading to the random coil conformation for pHly-1 and formation of small-sized nanoparticles because of electrostatic repulsion among the peptides (state I). Upon binding to the surface of bacterial membranes, the peptide undergoes a lipid-responsive coil-helix transition and penetrates into the bilayer (state II) as a typical amphiphilic α-helical cationic antimicrobial peptide. Therefore, under the acidic condition and binding with bacterial lipid bilayers, pHly-1 exhibits strong antimicrobial activity as an amphiphilic helical structure, which is the canonical conformation causing membrane disruption. In contrast, at neutral pH, the imidazole groups of histidine residues tend to be neutral and the peptide preferentially forms intermolecular hydrogen bonds, leading to the *β*-sheet conformation for pHly-1. The *β*-sheets then assemble into nanofibers through hydrogen bonding and hydrophobic interactions (Figure S4), transforming into large aggregates (state III). The bundled nanofibers might limit the penetration of peptide pHly-1 into the bacterial membrane bilayer, thus exhibiting low activity at neutral pH for pHly-1.

### pHly-1 NPs show rapid killing activity against clinical strains under acidic conditions

To further evaluate the bacterial killing potential of pHly-1 NPs, 20 clinical *S. mutans* strains from different age groups were isolated. The experimental protocol for isolation, identification, and treatment of clinical *S. mutants* strains is depicted in Figure S5. The MIC values of pHly-1 NPs or CHX against 20 bacterial strains were estimated to be the range from 5.5 to 11 μM, or 0.5 to 2 μM, respectively, at pH 5.5 depending on the strain types (Table S2). These results indicate the stronger bacteriostatic ability of CHX than pHly-1 NPs. In contrast, at pH 4.5 or 5.5, pHly-1 NPs exhibited an advanced bacterial killing performance compared to CHX as illustrated in Table S3. For example, incubation of bacteria in the presence of 55 μM pHly-1 NPs led to over 90% of bacteria death in 15 clinically isolated strains at pH 4.5. However, treatment with 55 μM CHX only showed its bactericidal ability for 19 strains with >50% and 4 strains with >90% viability. Also, increasing the concentration of pHly-1 NPs or CHX caused an improved reduction of bacterial viability. Overall, our experimental results clearly demonstrate that pHly-1 NPs exhibit a pH-dependent killing effect and show a stronger activity at pH 4.5 compared to pH 5.5, whereas the bacterial killing ability of CHX is pH-independent.

### Mechanistic analysis antibacterial activity of pHly-1 NPs

Direct disruption of bacterial membranes is the most conventional mechanism responsible for the bacterial killing by most AMPs 34,35. We found that treatment bacteria with pHly-1 NPs triggered acid-activated membrane disruption as revealed by bacterial morphological and fluorescence staining studies. Upon treatment of *S. mutans* UA159 with 22 μM pHly-1 NPs at pH 4.5 or 7.0 for 1 h, scanning electron microscopy (SEM) image illustrated the fragmentation of bacterial membrane and the intact membrane at pH 4.5 or 7.0, respectively, indicative of the membrane disruption by pHly-1 NPs at pH 4.5 (Figure 3A). In contrast, the morphology of bacteria was not affected by CHX regardless of pH conditions (Figure 3A). The bacterial membrane damage induced by pHly-1 NPs at pH4.5 was confirmed by SYTO 9/PI staining assays, in which dead bacteria with damaged membranes or living bacteria with intact membranes are stained in red and green, respectively. As shown in Figure 3B, the red fluorescence signals were only observed in *S. mutans* UA159 treated with 22 μM pHly-1 NPs at pH 4.5. These results indicate the acid-activated membrane disruption by pHly-1 NPs, which potentially contributes to the fast bacterial killing effect of pHly-1 NPs under the acidic condition.

Transcriptomics analysis revealed that treatment of *S. mutans* UA159 with 22 μM pHly-1 NPs and incubation at pH 4.5 for 1 h caused apparent alteration of gene expression as evidenced by the 195 differentially expressed genes (DEGs) between the control and peptide-treating groups analyzed by high-throughput sequencing (Figure S6). Subsequently, gene functional annotation enrichment analysis of DEGs showed top 15 terms mainly involved in protein folding, programmed cell death, cytolysis, transcription regulation, and biosynthesis including glucose, amino acids and isoprenoids (Figure 3C), which play important roles in bacterial survival and growth. Furthermore, as shown in Figure 3D, we found that pHly-1 NPs treatment led to increased expression of the genes regulating cytolysis, consistent with the lethal effect of pHly-1 NPs on bacteria under acidic conditions. Also, the treatment caused the lowered expression of the genes regulating glucose metabolism, nucleic acid metabolism, and DNA replication. In additional to the disrupted bacterial membranes, the gene expression data suggested that treatment with pHly-1 NPs might regulate the expression of the genes associated with antimicrobial activity. Interestingly, pHly-1 NPs treatment down-regulated quorum sensing-related genes, which are associated with bacterial proliferation and group behavior regulation during development of the bacteria biofilms 36. This process of quorum sensing might account for the inhibiting effect of pHly-1 NPs on the bacterial biofilm formation and development described below.

### pHly-1 NPs inhibit *S. mutans* biofilm formation and development *in vitro*

It has been reported that AMPs exhibit inhibitory activity on pathogenic biofilm formation and development, which is essential for treating biofilm-induced diseases 19. Dental caries is a biofilm-induced disease, and *S. mutans* is a broadly recognized biofilm-forming cariogenic pathogen. We first determined the minimum biofilm inhibition concentration (MBIC_50_) of pHly-1 NPs and CHX against *S. mutans* UA159. As shown in Figure S7, MBIC_50_ values of pHly-1 NPs and CHX were estimated to be 6.7 μM and 0.6 μM, or >22 μM and 0.8 μM at pH 5.5 and 7.0, respectively. The bacterial attachment in the initial stage of biofilm formation was inhibited and the bacterial clusters of biofilms was also strongly suppressed by pHly-1 NPs due to their antibacterial activity (Figure S8).

**Figure 4:**
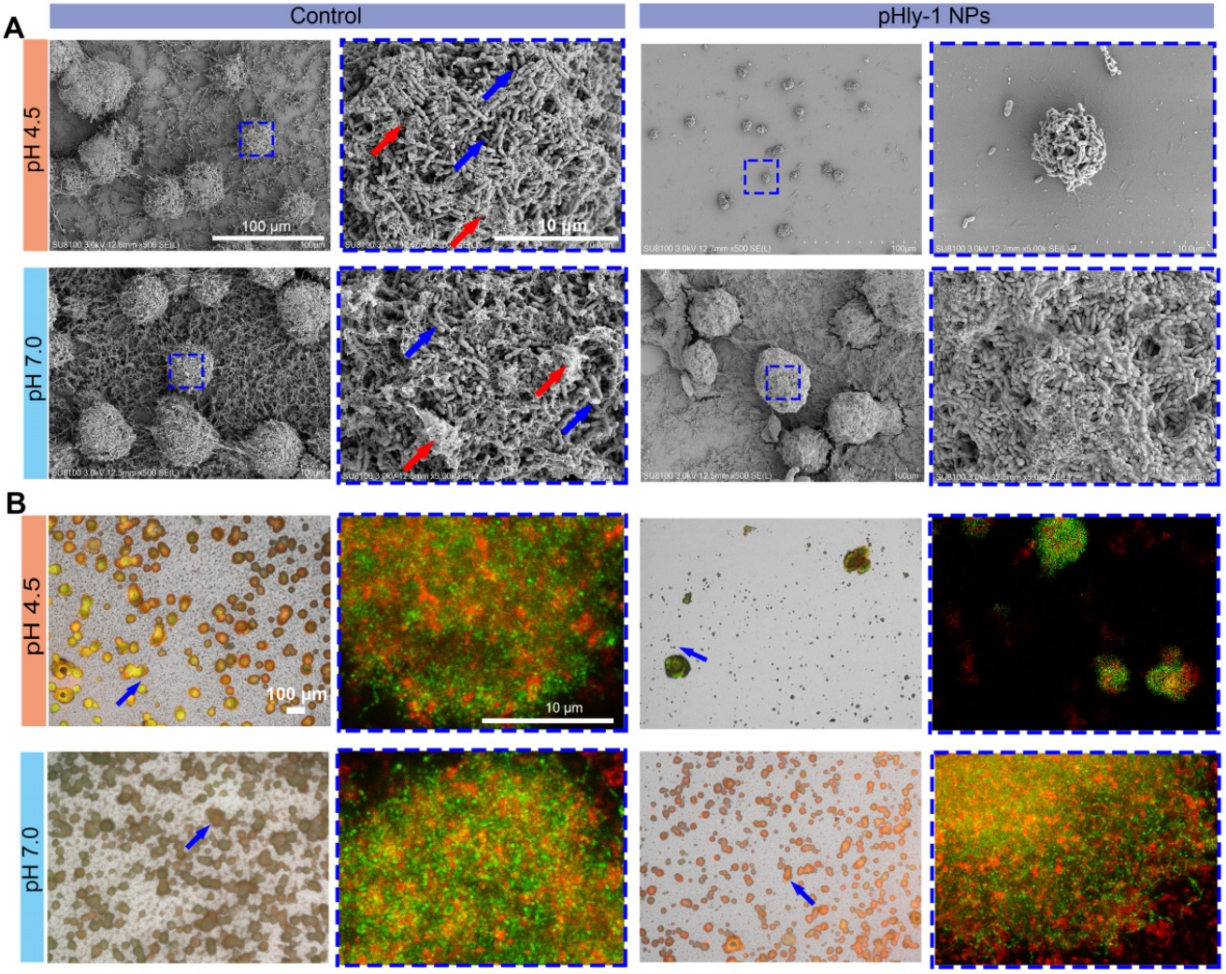
** Inhibition of *in vitro* biofilm formation and development of *S. mutans* UA159 by pHly-1 NPs at acidic conditions.** The biofilms were formed on circular glass slides for 12 h, and then treated with 55 µM pHly-1 NPs at pH 4.5 or 7.0 for 10 min, each every 12 h for 3 times in total. **(A)** SEM images of the biofilms of the control and pHly-1 NPs treated groups under different pH conditions. Blue box indicates the selected area for zoomed-in images of bacteria and EPS components. Blue arrow: *S. mutans* UA159 cells; red arrow: EPS components. **(B)** Confocal images of biofilms of the control and pHly-1 NPs treated groups under different pH conditions. Blue arrow and box indicate selected area for zoomed-in images of bacteria and EPS components. The *S. mutans* UA159 cells and EPS were colored in green by SYTO 9 or in red by Alexa Fluor 647-dextran conjugate, respectively.

Furthermore, we sought to evaluate the potency of pHly-1 NPs to inhibit the development of pre-formed biofilms. *S. mutans* UA159 was first cultured on glass slides in the presence of 1% sucrose, a substrate for the synthesis of the extracellular polymeric substance (EPS) matrix and gradual formation of biofilms on slides. After 12 h, 55 μM pHly-1 NPs or CHX was repeatedly loaded to the slides and incubated with the EPS for 10 min at different pH. The treating process by individual treatment was repeated each every 12 h in a total of 3 times. SEM images of *S. mutans* (blue arrow) in the control group showed the interconnected EPS matrix (red arrow) surrounding the bacteria and formation of a cohesive and densely packed microbial structure (Figure 4A). In the pHly-1 NPs treatment group at pH 4.5, the 'dome-shaped' bacterial clusters 12 were significantly smaller than in the control group. Likewise, the number of the bacteria and the density of the EPS matrix were also significantly lower than those of the control group (Figure 4A). However, these changes were not observed between the pHly-1 NPs and CHX treatment groups at pH 7 (Figure 4A and Figure S9A). These data were further validated by the quantitative analysis of the collected and homogenized biofilms (Figure S9B-C). The SYTO 9 and Alexa Fluor 647-dextran conjugate were used to label *S. mutans* and EPS on slides, and the biofilms were imaged by confocal microscopy. The bacterial clusters (in green) were spatially localized within the EPS matrix (in red) in the control groups, but pHly-1 NPs treatment at pH 4.5 caused significant inhibition of bacterial clusters and formation of EPS matrix (Figure 4B). This suppression effect was almost negligible for the bacteria treated with pHly-1 NPs at pH 7.0 (Figure 4B) or by CHX (Figure S10A-B). Therefore, these data showed that pHly-1 NPs could potentially inhibit the biofilm development of *S. mutans* in a pH-dependent manner.

### pHly-1 NPs inhibit human-derived *ex vivo* biofilm development

**Figure 5:**
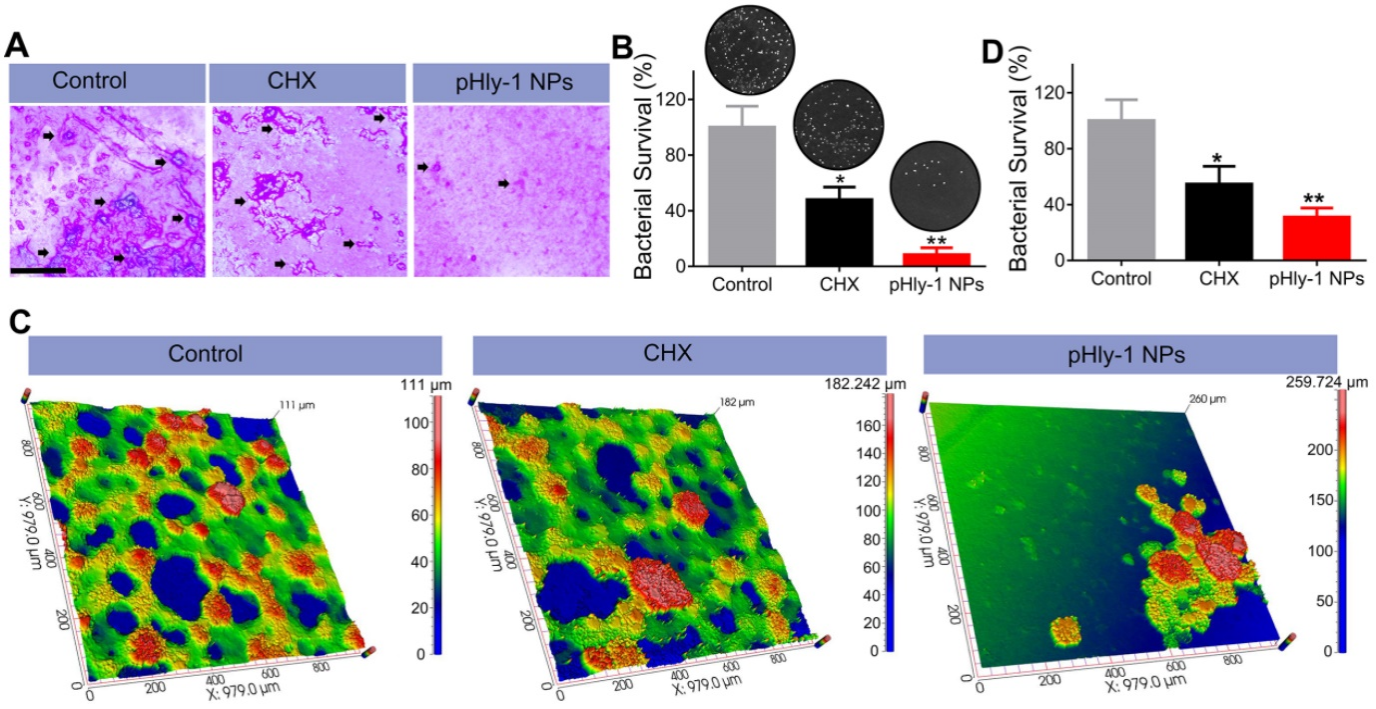
** pHly-1 NPs inhibits *ex vivo* biofilm development under the acidic condition. (A)** Optical microscopic images of the of* S. mutans* UA159 biofilms formed on sHA surfaces stained by crystal violet. Scale bar, 250 µm. The black arrows indicate the formation of *S. mutans* biofilms. **(B)** Quantitative analysis of the total viable cell count after treatment of the biofilms on sHAs with control, CHX and pHly-1 NPs, respectively (n = 3). **(C)** Optical 3D surface topographic images of the *ex vivo* biofilms formed on natural human tooth-enamel models. The biofilms were formed by the plaque-biofilm samples from four children with S-ECC, respectively.** (D)** Quantitative analysis of the total viable cell counting after treatment of the biofilms on natural human tooth-enamel with control, CHX and pHly-1 NPs, respectively (n = 4). The treatment protocol was shown in Figure S11. In brief, 24 h after bacteria seeding on the surfaces of sHA or natural human tooth-enamel, biofilms were treated with 55 µM pHly-1 NPs or CHX for 10 min at pH 4.5, once per day in a total of 5 days. One-way ANOVA with Tukey's correction was used for statistical analysis. Data are shown as mean ± s.d.

We further investigated the inhibition of biofilm development by pHly-1 NPs based on saliva-coated hydroxyapatite (sHA) and natural human tooth enamel models. The treatment regimen was adapted from the reported literature 12 (Figure S11). Since the negligible antibiofilm activity of pHly-1 NPs under the neutral condition, the treatment was only carried out at pH 4.5. As displayed in Figure 5A, bacterial colonies composed of biofilms on the sHA surfaces were stained by crystal violet (black arrow). Compared with the control and CHX-treated groups, pHly-1 NPs treatment greatly reduced the number and size of colonies on the sHA surface (Figure 5A). Quantitative analysis as determined by CFU counting confirmed the significantly lowered viability of the *S. mutans* after pHly-1 NPs treatment compared to the control group (Figure 5B). Unlike the *in vitro* studies where CHX did not show obvious inhibition on the biofilm development (Figure S9B), the *ex vivo* experiments revealed the apparent antibiofilm efficacy of CHX compared to the control, likely due to the great adsorption of CHX on biofilm surface.

Encouraged by the antibiofilm potency of pHly-1 NPs on the sHA model, we collected plaque-biofilm samples from four children with severe early childhood caries (S-ECC). An *ex vivo* human biofilm model was established based on natural human tooth enamel that could mimic biofilms formed on the teeth of dental caries patients. *S. mutans* from different plaque-biofilm samples were first isolated to ensure a similar *S. mutans* proportion (90%) for the total bacterial inoculum. The images of the *ex vivo* biofilm, collected by the optical 3D surface profilometer (Figure 5C), indicated that pathogenic bacteria proliferated rapidly on human tooth enamel surfaces and interweaved into a network (in green) in the control and CHX treatment groups. However, few bacterial networks were observed in the group treated with pHly-1 NPs. Furthermore, more dense microbial structures (in red) mainly composed of *S. mutans* and EPS matrix were found in the control than those in pHly-1 NPs and CHX treatment groups. Quantitative analysis of the bacterial viability of three groups further indicated that pHly-1 NPs significantly inhibited the *ex vivo* biofilm development under the acidic condition (Figure 5D).

### pHly-1 NPs suppress dental caries *in vivo*

Considering the remarkable* in vitro* and *ex vivo* antibiofilm effect of pHly-1 NPs, we further assessed whether pHly-1 NPs could suppress dental caries development *in vivo* using a well-established *S. mutans* infection model of rat pups that mimics the characteristics of severe early childhood caries 12. We established *S. mutans* infection on the teeth in 15-day-old female Sprague -Dawley rat pups. Subsequently, CHX or pHly-1 NPs were topically administrated on teeth (100 μM, 300 μL per animal) once per day. The effects of pHly-1 NPs and CHX on teeth were examined over 35 days after drug administration.

**Figure 6:**
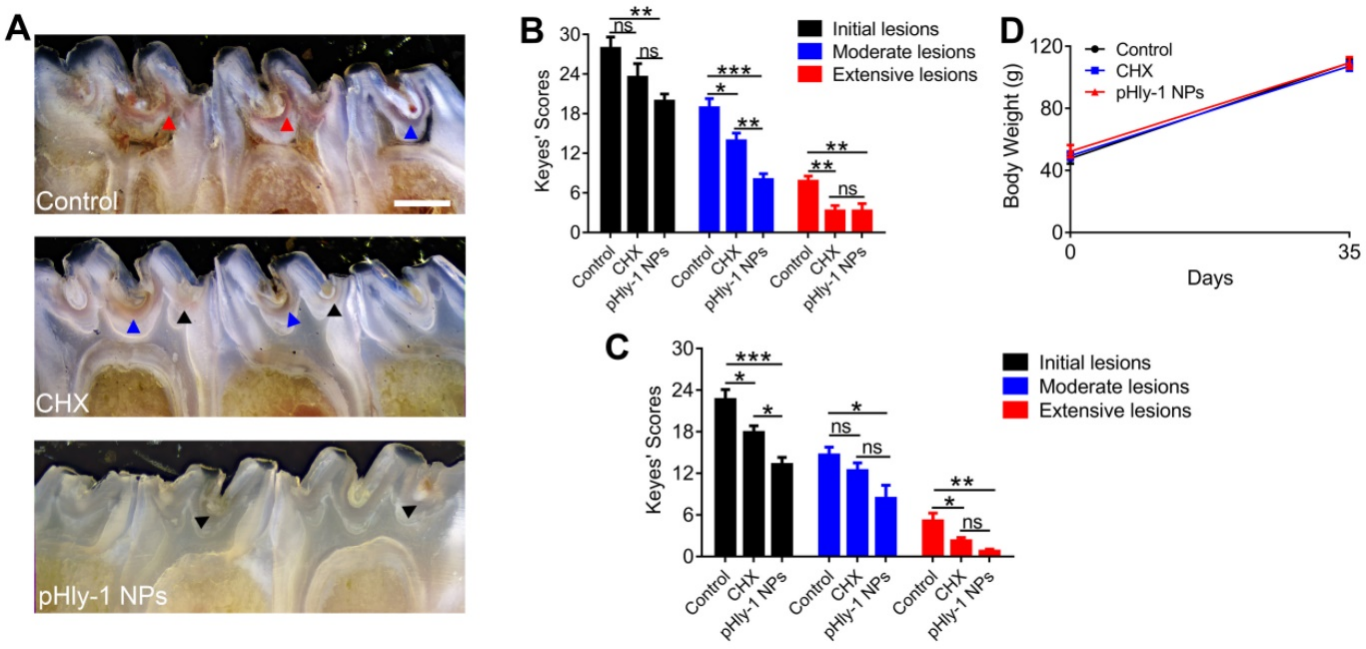
** Therapeutic efficacy of pHly-1 NPs against dental caries in rat pup model. (A)** Representative photographs of caries lesions at the 36^th^ day post topical treatment. Black arrows, blue arrows and red arrows indicate initial, moderate and extensive carious lesions, respectively. Scale bar, 1 mm. **(B)** Quantitative analysis of caries onset and severity of sulcal. **(C)** Quantitative analysis of caries onset and severity on smooth surfaces. **(D)** Body weight changes of rat pups within the duration of topical treatment. The rat pup dental caries model was established by infection of teeth with *S. mutans* UA159, and the topical application was lasting for 35 days. The concentration of pHly-1 NPs or CHX was 100 µM and pH was 4.5. Carious lesions were initially evaluated with murexide staining 38,39, and caries scores were recorded based on stages and extent of carious lesion severity according to Larson's modification of Keyes' scoring system. One-way ANOVA with Tukey's correction was used for statistical analysis. Data are shown as mean ± s.d, n = 8.

After the *S. mutans* infection, severe carious lesions were progressively developed on the teeth. Subsequently, murexide staining of tooth slices was used to evaluate carious lesions. Figure 6A displayed the representative photographs of tooth slices from rat pups treated with control, CHX, or pHly-1 NPs. The smooth and sulcal surfaces were stained in deep brown in the controls, indicating that *S. mutans* infection induced moderate (blue arrows) and severe (red arrow) lesions. On the other hand, in the groups treated with CHX or pHly-1 NPs, both smooth and sulcal surfaces were lightly stained, showing initial (black arrows) or moderate (blue arrows) lesions. In particular, teeth in the pHly-1 NPs treatment group appeared nearly normal, suggesting the prevented development of caries by pHly-1 NPs even after *S. mutans* infection of the teeth. The caries severity was analyzed according to Larson's modification of Keyes' scoring system 37 (Figure 6B-C). Compared with the control, pHly-1 NPs treatment significantly decreased the occurrence of initial, moderate, and severe lesions in both sulcal (Figure 6B) and smooth surfaces (Figure 6C). Especially, severe lesions were inhibited to a very low level upon the treatment with pHly-1 NPs. Similarly, CHX treatment also alleviated severe lesions, but, as expected, did not yield good outcomes for initial and moderate lesions. It is noteworthy that pHly-1 NPs exhibit a better therapeutic effect than CHX. No significant difference in body weights was found between the control and treatment groups after the treatment period of 35 days, indicating the excellent biosafety of pHly-1 NPs and CHX towards the rats (Figure 6D). Furthermore, hematoxylin and eosin (H&E) staining showed that compared with controls, pHly-1 NPs and CHX presented no detectable signals for the harmful effect on the mouse gingival, palatal and gastric tissues (Figure S12).

### *In vitro* toxicity analysis of pHyl-1 NPs

**Figure 7:**
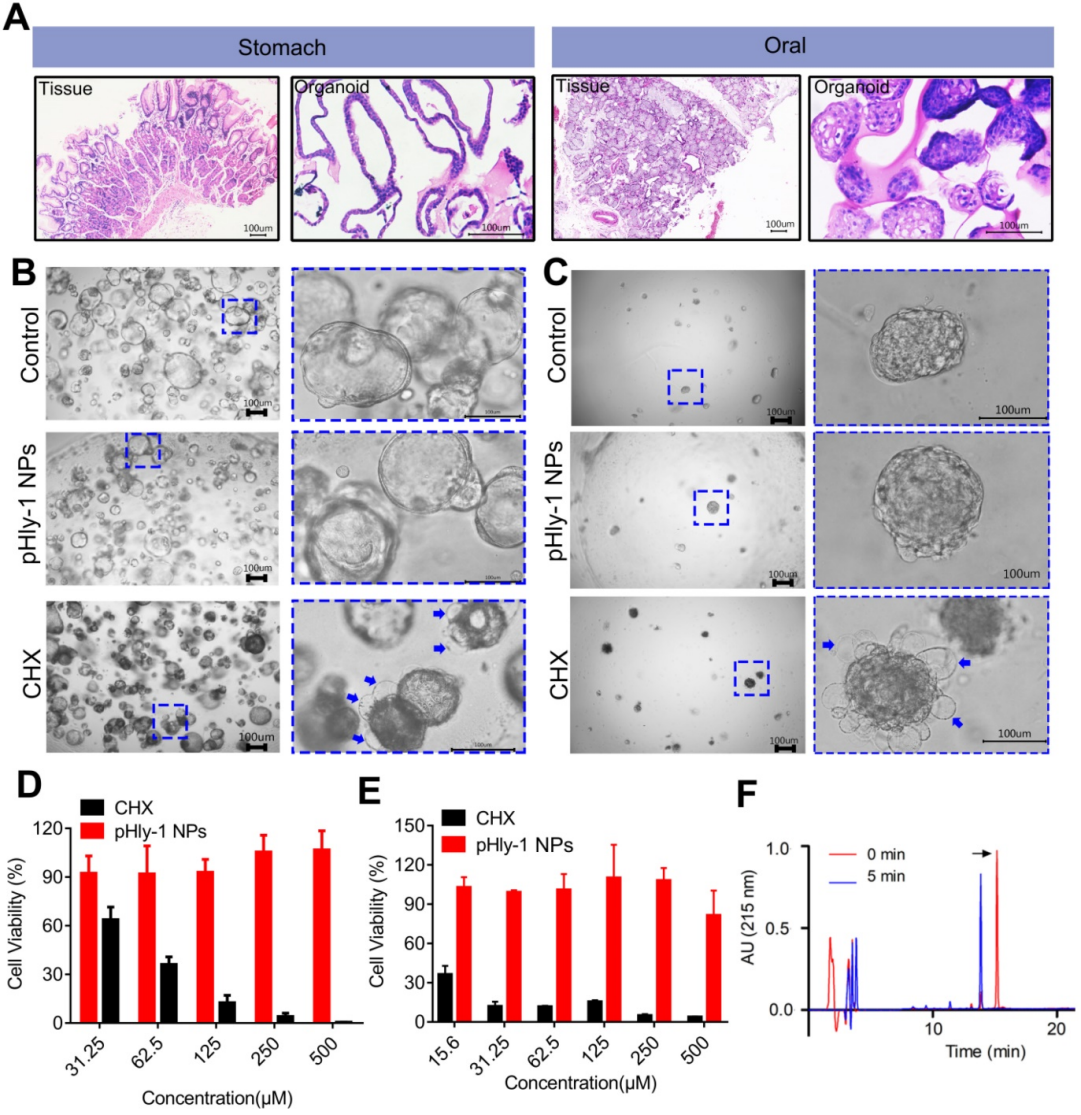
** The biosafety of pHly-1 NPs to normal oral and gastric organoids. (A)** H&E staining revealed notable morphological similarities between oral/gastric organoids and oral/gastric tissue collected from patients. **(B and C)** The representative gastric organoid images (**B**) and oral organoid images (**C**) after 500 µM pHly-1 NPs or CHX treatment. **(D and E)** the viability of gastric (**D**) and oral (**E**) organoids after treatment with pHly-1 NPs or CHX was assessed by CellTiter-Blue cell viability assay. Scale bar, 100 µm. Each experiment was repeated 3 times. (**F**) RP-HPLC analysis of pHly-1 NPs degradation by pepsin. pHly-1 NPs (100 µg/mL) was incubated with pepsin (10 µg/mL) in Tris buffer (pH 4.0) at 37 °C for 5 min and submitted to RP-HPLC analysis. The HPLC fractions were analyzed by MALDI-TOF MS. The black arrow represents the intact peptide of pHly-1 NPs and the molecular weight is 2865.1 Da (Figure S14). After 5 min of pepsin hydrolysis, the molecular weight of the mainly peptide fragment is 1650.6 Da (Figure S14). All of the data are represented as average ± s.d.

The cytotoxic activity of pHly-1 NPs against human gingival fibroblast (HGF-1) was assessed by the CCK-8 assay, showing the IC_50_ values of 4.9 μM and >500 μM for CHX and pHly-1 NPs, respectively. However, normal organoids derived from patients are better to represent native tissues and may be the superior models to test the *in vitro* toxicity of drugs. Therefore, we used the normal oral and gastric organoids established in our laboratory to further evaluate the toxicity of pHly-1 NPs. While pHly-1 NPs had no apparent effect on both organoids, organoid death or decreased viability was induced by treatment with ~20 μM CHX. Especially, H&E staining revealed notable morphological similarities between oral/gastric organoids and gastric tissues from patients (Figure 7A). The gastric organoids showed cystic structures in the control and pHly-1 NPs treatment groups. When incubated with CHX, gastric organoids displayed dense morphology and bubbles (blue arrow) around the organoids (Figure 7B). As shown in Figure 7D, the viability test indicated that 31.25 μM CHX induced about 40% of gastric organoids death, but no obvious effect was observed with 500 μM pHly-1 NPs treatment. The oral organoids demonstrated dense structures in the control and pHly-1 NPs treatment groups; however, following the CHX treatment, numerous bubbles (blue arrow) around the organoids indicated cell death (Figure 7C). The viability test showed that 15.6 μM CHX induced about 60% of oral organoid death; however, no apparent viability decrease was detected by 500 μM pHly-1 NPs treatment (Figure 7E).

Orally administrated pHly-1 NPs could exhibit toxicity to gastric tissues due to acid activation in the stomach. However, peptides are usually not stable and lose activity due to their rapid digestion by endogenous proteases. After incubation with the main digestive protease in the stomach pepsin at pH 4.0 for 5 min, pHly-1 NPs were rapidly degraded as detected by HPLC analysis (Figure 7F and Figure S14). These results may indicate the low toxicity of pHly-1 NPs on gastric tissues even under acidic conditions, effectively avoiding potential gastric damage caused by the previously reported dental caries prevention nanoparticles 6.

### Analysis of adverse effect of pHly-1 NPs on oral microbiota and surrounding tissues

CHX is commonly used in antiseptic mouthwashes; however, its utilization in a long term inevitably results in toxicities, such as disturbing oral microbiota diversity and injuring oral and gastric tissues 40,41 We conducted assays using Kuming mice to assess the safety of pHly-1 NPs and CHX according to the previously described CHX preclinical safety evaluation method 41. Aqueous solution of pHly-1 NPs or CHX at a concentration of 0.2% in 100 μL volume was gradually added to the mouth of mice once per day for 7 days. On the 8^th^ day, saliva was drawn for oral microbiome analysis, and gingival, palatal, and gastric tissues were collected for histopathological analysis. Adverse effect was observed in the CHX treatment group, rather than in the pHly-1 NPs treatment group.

**Figure 8:**
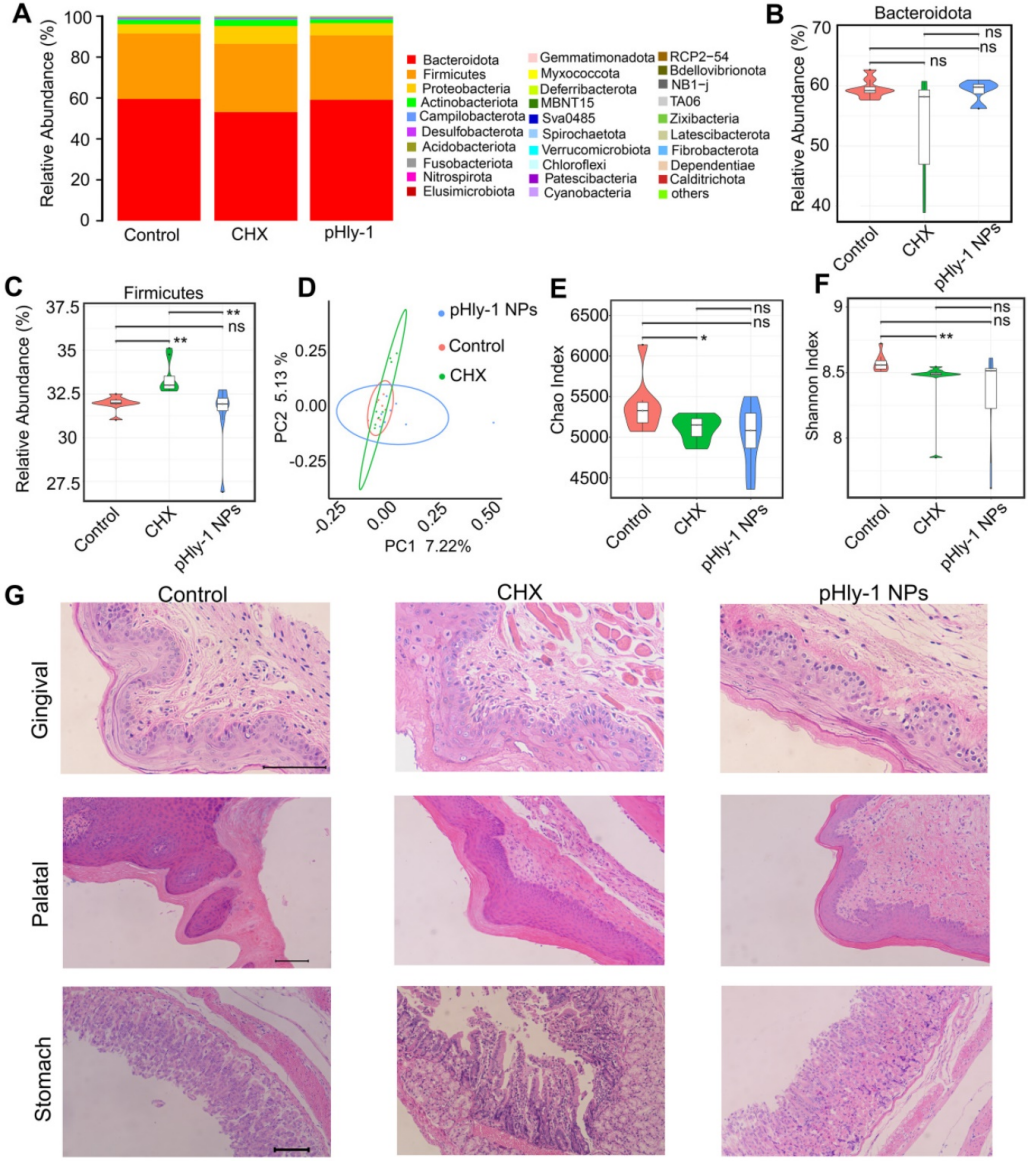
** Adverse effect analysis of pHly-1 NPs on oral microbiota and surrounding tissues. 0.2 % pHly-1 NPs or CHX of 100 µL was continuously dropped to mouse mouth once a day for 7 days in total.** At the 8^th^ day, saliva was drawn for oral microbiome analysis, and gingival, palatal and gastric tissues were collected for histopathological analysis. **(A)** Relative abundance of the top thirty bacterial phyla. **(B and C)** The statistics on the top two phyla Bacteroidetes (**B**) and Firmicutes (**C**). (**D**) Weighted Unifrac principal coordinate analysis (PCoA) representing beta-diversity after a 7-day treatment with drugs. **(E and F)** Chao's (**E**) and Shannon's (**F**) index representing alpha-diversity. Wilcoxon test was used for statistical analysis. Data are shown as mean ± s.d. (n = 8). **(G)** H&E staining of gingival, palatal and gastric tissues. Scale bar, 100 µm.

The results from oral microbiome analysis were displayed in Figure 8, which showed alteration of the entire phyla by CHX but not pHly-1 NPs (Figure 8A). Statistical analysis of the top three phyla (Bacteroidetes, Firmicutes and Proteobacteria) indicated that pHly-1 NPs did not affect their abundance. On the contrary, CHX significantly increased the abundance of Firmicutes species and had a tendency to lower Bacteroidetes species (Figure 8B-C and Figure S15A), thereby increasing the ratio between the two main phyla (Firmicutes/Bacteroidetes) (Figure S15B). This increment may decrease saliva pH and its buffering capacity, promoting the development of dental caries as reported in the literature 41. The relative abundance of the main genus in the pHly-1 NPs treatment group was comparable with the control group, indicating their similar oral microbial composition (Figure S15C). As expected, pHly-1 NPs could efficiently suppress oral disease without affecting the oral microbiota composition. However, CHX affected the composition of oral flora, such as increasing Streptococcus and decreasing Muribaculaceae (Figure S15C). The principal coordinate analysis demonstrated a greater similarity between pHly-1 NPs and the control than CHX and the control, indicating that pHly-1 NPs did not disrupt the ecological balance of the microbiota (Figure 8D). Also, the Chao and Shannon analysis index indicated that CHX but not pHly-1 NPs reduced the oral microbiota diversity and therefore disrupted the ecological balance of the microbiota (Figure 8E-F). Moreover, H&E staining showed no visible signs of harmful effects on the mouse gingival, palatal, and gastric tissues by pHly-1 NPs (Figure 8G). On the contrary, epithelial cell shedding and structural damage of gastric tissue were observed in the CHX treatment group, indicating CHX had a potential risk of impairing stomach tissues after entering the stomach following oral administration, However, it did not have an apparent damaging effect on gingival and palatal tissues. Furthermore, tooth staining, one of the adverse effects of CHX, was not observed in the pHly-1 NPs treatment group but was occasionally present in the CHX treatment group (data not shown).

## Discussion

Peptide nanotechnology offers tremendous possibilities to overcome the adverse effects and improve pharmacological properties via various stimuli-responsive systems design. Acidic biofilm microenvironment mediated mainly by *S. mutans* plays key roles in carcinogenesis and dental caries development 4. Targeting the microenvironment could be a feasible strategy for therapeutic drug development with high safety and efficacy 42,43. In this study, we designed an anticarious AMP named pHly-1 based on the lycosin-I sequence, a well-studied AMP in our laboratory. pHly-1 was obtained by an optimization process including histidine residue introduction, charge change, and hydrophobicity adjustment through amino acid residue substitution. Our study indicated that pHly-1 possessed self-assembling properties and exhibited different nanostructures in response to pH changes. Importantly, pHly-1 was only functional upon binding to membrane lipids, and underwent a conformational transformation in acidic but not neutral conditions. Thus, the activity of pHly-1 was acid- and lipid-responsive. Under acidic conditions, pHly-1 NPs demonstrated inhibitory activity and rapid bactericidal effect on *S. mutans,* including clinically isolated *S. mutans* strains from patients. Furthermore, pHly-1 NPs suppressed the formation and development of biofilms in the *in vitro* and *ex vivo* models mimicking cariogenic biofilms of human patients. However, pHly-1 NPs did not affect *S. mutans* and mouse red cells under neutral conditions. Significantly, pHly-1 NPs could efficiently prevent and treat dental caries in the *S. mutans*-induced rat pups carious model with no noticeable adverse effects.

The charged states of histidine residues under different pH conditions control the net positive charge and conformation of the pHly-1, which might be the mechanism of the pH- and lipid-sensitive activity of pHly-1. As an acid-sensitive element, histidine is widely used for pH environment adaptability in drug design 44-48. For example, histidine residues provide a mechanism to control the assembly and disassembly of peptide C8 based on acid sensitivity, resulting in its selective anticancer activity in the tumor acidic microenvironment 49. A naturally occurring peptide clavanin A, rich in histidine and phenylalanine residues with pH-dependent antimicrobial activity, serves as a design basis for these prototype “acid-activated peptides” (AAPs) 50. In our study, under neutral conditions, most histidine residues of pHly-1 are deprotonated, enable different side chain interactions e.g., hydrophobic and hydrogen bonds, and are alternatively arranged (Figure S4), leading to paired side chain interactions which might allow the formation of *β*-sheets and facilitating the stacking of *β*-sheets. The *β*-sheet stacks are usually prone to aggregation, impeding the insertion of pHly-1 across the bacterial membrane bilayer and depriving it of antimicrobial activity. However, further application of pHly-1 NPs is limited by the formation of large aggregates even precipitation. Therefore, further peptide optimization, such as PEG modification, will be our next research focus to solve this limitation of peptide aggregation. At acidic conditions, most histidine residues are protonated, which would provide pHly-1 with high electro-positivity and strong electrostatic repulsion, resulting in their binding with membrane lipids. pHly-1 exhibited lipid-responsive coil-helical conformation transition with potent membrane disruption capability. Predictably, due to its pH- and lipid-responsive antimicrobial activity, pHly-1 might have broader applications in other pathological conditions with acidic pH microenvironments, such as for the treatment of *Staphylococcal* infections 51,52.

AMPs are considered promising candidates for treating biofilm development- associated oral infections, including caries and periodontitis 53. Antibiofilm strategies mediated by AMPs might be involved in interfering with bacterial membrane integrity and potential, the rapid killing of bacteria and inhibiting bacterial quorum-sensing 19,53,54. Our studies revealed that pHly-1 NPs shared the common mechanism of antibiofilm activity with most AMPs. Bacterial membrane integrity and potential play important roles in biofilm formation and development. At the initial stage of biofilm formation, bacteria gather continuously and reversibly on the surface through weak interactions, such as van der Waals and electrostatic forces 20,55. Reduced bacterial membrane integrity may impair bacterial adhesion and suppress biofilm formation. Interfering with bacterial membrane integrity might inhibit biofilm development by impairing interactions between EPS and bacteria at the later stages of established biofilms. For example, the designed helical peptide G3 decreased the membrane integrity and surface charges of *S. mutans* to inhibit the initial bacterial aggregation or attachment 56. Another example is Esculentin-1a which disrupted the biofilms of *Pseudomonas aeruginosa* via perturbing the extracellular matrix and destroying bacterial membranes 57. Similarly, our study demonstrated that pHly-1 NPs could interfere with the bacterial membrane integrity and potential by rapid disruption of *S. mutans* membranes and inhibit initial bacterial aggregation or attachment and EPS-bacteria interactions, resulting in the suppression of biofilm formation and development. Also, the rapid bactericidal ability is crucial for the antibiofilm activity of pHly-1 NPs to quickly reduce the density of* S. mutans,* a major producer of EPS, and also decrease EPS generation. *S. mutans* and EPS are the two key components in the carious biofilm structure. Furthermore, as mentioned above, quorum-sensing positively participates in the regulation of biofilm development. It has been reported that LL-37 could modulate the two main quorum-sensing systems of Las and Rhl to influence biofilm development 58. Our transcriptomic analysis demonstrated that pHly-1 NPs could attenuate the expression of some genes associated with quorum-sensing systems, suggesting their involvement in the antibiofilm activity of pHly-1 NPs.

We elucidated the anticarious potential of pHly-1 NPs, by performing a series of comparative experiments between pHly-1 NPs and CHX, the most widely used anticarious agent. CHX exhibited stronger bacteriostatic ability than pHly-1 NPs based on their MIC values. However, because of its strong bacterial membrane disruption activity, pHly-1 NPs killed bacteria faster and more efficiently than CHX. The rapid bactericidal activity is crucial to suppressing the development of biofilms attached to teeth, especially when drugs are administrated topically for a short duration. pHly-1 NPs exhibited a much higher efficacy than CHX for inhibiting *S. mutans* biofilm formation and development *in vitro* and *ex vivo*. Significantly, a much better therapeutic effect of pHly-1 NPs than CHX was observed in the rat carious model mimicking the severe early childhood caries. By contrast, the relatively weak antibiofilm ability of CHX is a limitation to its effectiveness as an anti-caries drug* in vivo*. Moreover, CHX, like some other antimicrobial agents, can even enhance the mechanical stability of biofilms, further undermining its *in vivo* efficacy 12. Also, CHX potentially impairs the ecological balance of oral microbiota and gastric tissues, whereas pHly-1 NPs, due to their acid-activated activity, exhibited higher safety with no evident side effects on rats.

## Conclusion

We have developed pH-sensitive AMP pHly-1 that undergo conformational and morphological transitions driven by pH changes. pHly-1 have advantages over CHX, exploit the acidic cariogenic biofilm microenvironment, and exhibit effective and safe performance to prevent and treat dental caries. This peptide design strategy may herald new avenues for improving the biocompatibility of AMPs and enabling their application in antimicrobial drug development.

## Supplementary Material

Supplementary figures and tables.

Supplementary methods.
