# Supplementary material for: Dual-sensitive antibacterial peptide nanoparticles prevent dental caries
Source: Theranostics. 2022 Jun 13;12(10):4818–33. doi: 10.7150/thno.73181 (PMC9254247; doi:10.7150/thno.73181)
Supplement: Supplementary file 1 — Supplementary figures and tables. [file thnov12p4818s1.pdf]

## Supplementary figures

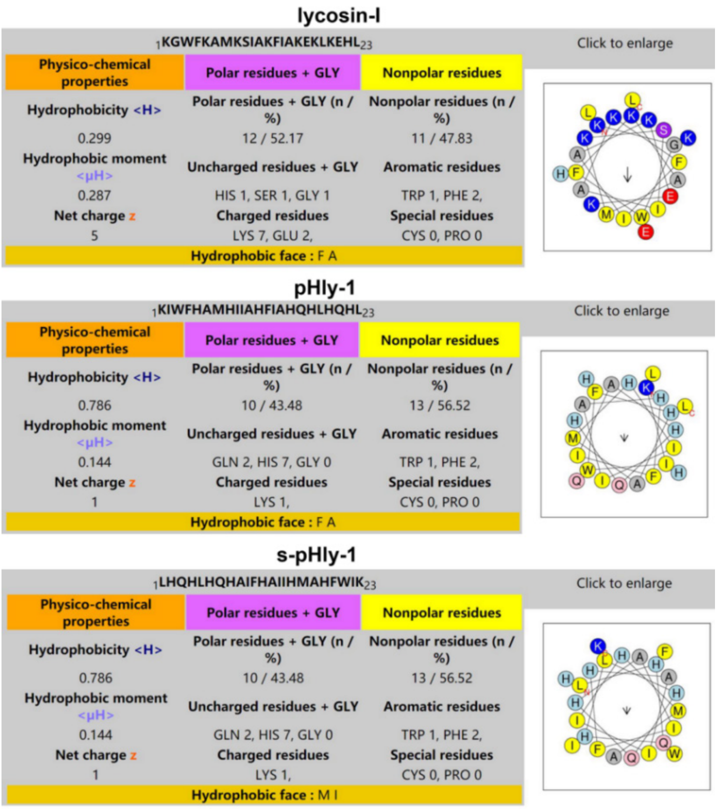


**Supplementary Fig.1. Helical wheel structures of peptides we designed were predicted by heliquest** (https://heliquest.ipmc.cnrs.fr/cgi-bin/ComputParams.py).

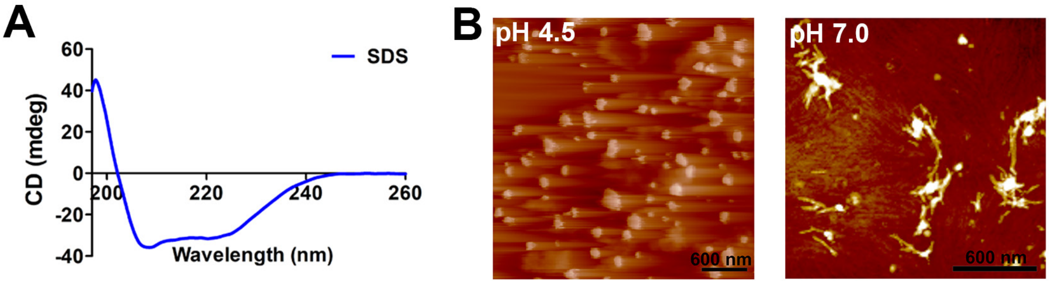


**Supplementary Fig.2. Physicochemical characterization of pHly-1 NPs at pH 4.5 and 7.0. A**, The CD spectra of pHly-1 NPs at pH 7.0 in SDS. **B**, AFM images of the pHly-1 NPs at pH 4.5 and 7.0, respectively.

**Table S1 MIC, $MIC_{50}$ and $HC_{50}$ of peptides**

| Peptide | MIC (μM) | | $MIC_{50}$ (μM) | | $HC_{50}$ (μM) |
|---|---|---|---|---|---|
| | pH 5.5 | pH 7.0 | pH 5.5 | pH 7.0 | |
| lycosin-I | 44 | ~ 44 | 21.9 | 31.7 | 180 |
| pHly-1 NPs | 5.5 | >44 | 2.8 | 15.6 | >500 |
| s-pHly-1 NPs | >44 | >44 | 26.0 | >44 | 423.0 |

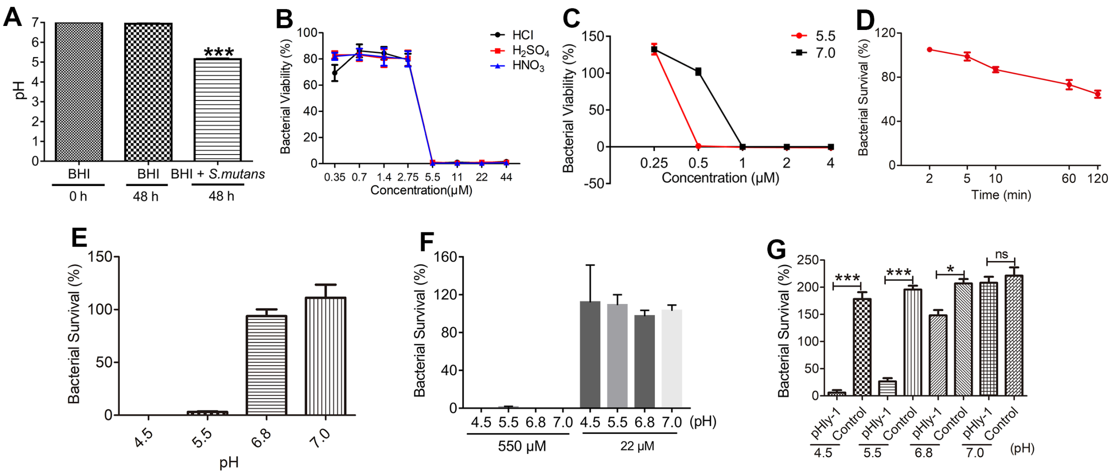


**Supplementary Fig.3. The pH of BHI medium and the bacterial killing activity of CHX and pHly-1. A**, The determination of the pH of BHI medium. The initial pH of BHI medium was 7.0. After 48 hours of culture, the pH of the medium did not change; but the pH of medium contained *S.mutans* UA159 (1× $10^6$) decreased, which might be related to the acid-producing property of *S.mutans* UA159. B, The MIC was also performed when acidic BHI medium is modulated with different acids including HCl, $H_2SO_4$ and $HNO_3$. C, The MIC values of CHX against *S.mutans* UA159. The bacteria were incubated with series concentrations of CHX at pH 5.5 or 7.0 for 48 h. D, The time-killing kinetics of CHX against *S.mutans* UA159. The bacteria were treated by CHX at the concentration of 22 μM (4 MIC). E, The killing activity of 22 μM pHly-1 NPs was measured against *S.mutans* UA159 at pH 6.8 and 7.0, and the killing activity of 550 μM pHly-1 NPs was measured against *S.mutans* UA159 at pH 4.5 and 5.5, which all as revealed by counting the CFU of alive bacteria after 10 min incubation.F, The killing activity of 22 and 550 μM CHX was measured against *S.mutans* UA159 at different pHs as revealed by counting the CFU of alive bacteria after 10 min incubation. G, In this *in vitro* experiment only, the stock solutions of pHly-1 was PBS (4.5). The bacteria were treated with 22 (4 MIC) or 550 μM (100 MIC) of pHly-1 for 10 min under acidic conditions (4.5 and 5.5) and neutral conditions (6.8 and 7.0), respectively. All of the data are presented as average± SD. Each experiment consists of 3 repetitions.

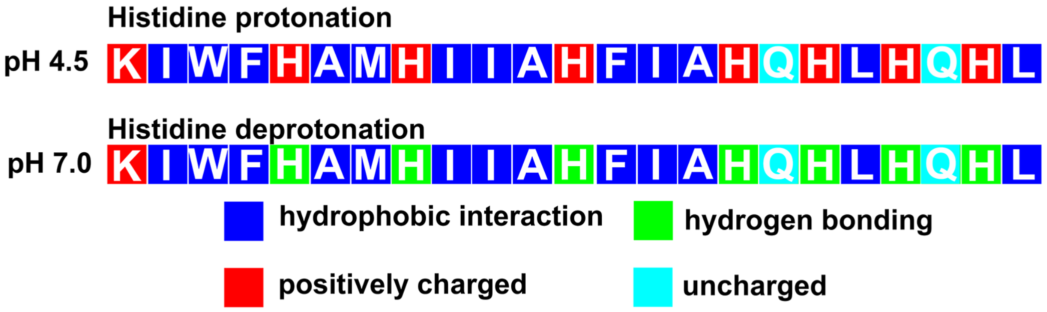


**Supplementary Fig.4. The overall intermolecular forces analysis depending on the protonation status of histidine residues.** The colors represent different types of forces provided by the side chains of amino acids. When the histidine residues were protonated, the overall intermolecular forces could become repulsive, leading to unstructured random coiled conformation. When the histidine residues were deprotonated, the overall intermolecular forces could become attractive due to intra-nanofiber hydrogen bonding and hydrophobicity interaction, which may cause fiber to tend to aggregate.

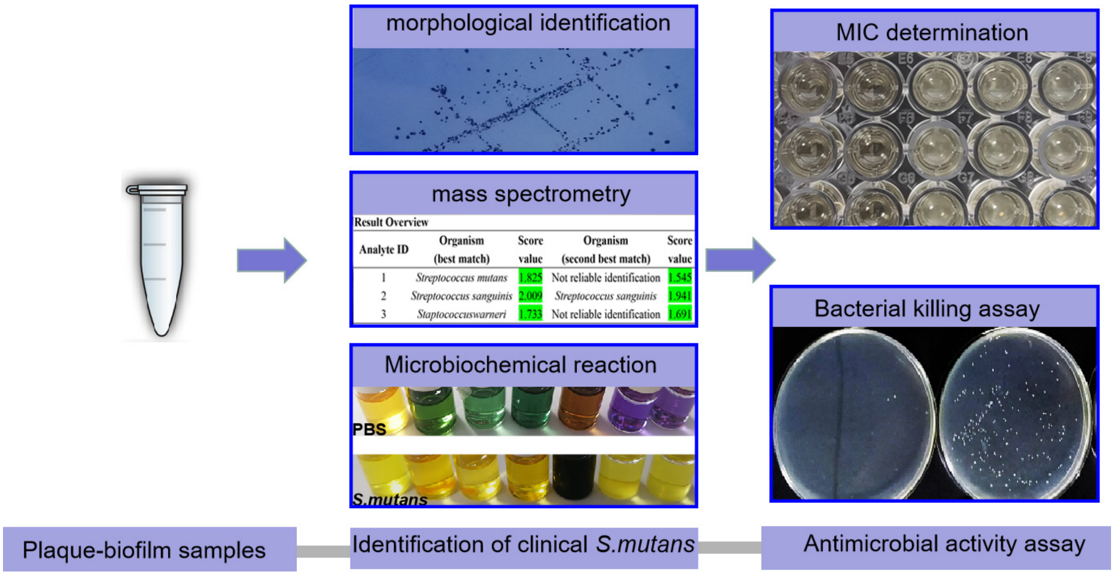


**Supplementary Fig. 5. Experimental design for isolation, identification and treatment of clinical *S.mutans* strains.** Plaque biofilm samples were collected from 20 patients. After isolation, the *S.mutans* were preliminarily selected according to their morphological and further confirmed by the mass spectrometry and microbiochemical reaction. The isolated *S.mutans* were used as subjects to test the antimicrobial activity of pHly-1 NPs and CHX. Moreover, samples from 4 children were also used to establish the human-derived *ex vivo* biofilm model.

**Table S2 The MICs of pHly-1 NPs and CHX against clinically isolated *S.mutans* strains**

| strains[a] | MIC (μM) at pH 5.5 | |
|---|---|---|
| | pHly-1 NPs | CHX |
| CS-1 | 11[b] | 1 |
| CS-2 | 11 | 0.5 |
| CS-3 | 5.5 | 1 |
| CS-4 | 11 | 1 |
| CS-5 | 11 | 2 |
| CS-6 | 5.5 | 1 |
| CS-7 | 5.5 | 1 |
| CS-8 | 11 | 2 |
| CS-9 | 11 | 2 |
| CS-10 | 5.5 | 1 |
| CS-11 | 11 | 1 |
| CS-12 | 11 | 0.5 |
| CS-13 | 5.5 | 1 |
| CS-14 | 11 | 1 |
| CS-15 | 11 | 2 |
| CS-16 | 5.5 | 1 |
| CS-17 | 5.5 | 1 |
| CS-18 | 11 | 2 |
| CS-19 | 11 | 2 |
| CS-20 | 11 | 2 |

a: CS1-16 were isolated from the plaque-biofilm samples of middle-aged and young patients and CS17-20 were isolated from the plaque-biofilm samples of 4 children diagnosed with severe early childhood caries (S-ECC). Each experiment consists of 5

repetitions.

**Table S3 The bactericidal activity of pHly-1 NPs and CHX against clinically isolated *S.mutans* strains[a]**

| strains | pHly-1 NPs | | | | CHX | | | |
|---|---|---|---|---|---|---|---|---|
| | pH=5.5 | | pH=4.5 | | pH=5.5 | | pH=4.5 | |
| | 22 μM | 55 μM | 22 μM | 55 μM | 22 μM | 55 μM | 22 μM | 55 μM |
| CS-1 | 0 % | 0 % | 1.4 % | 3.1 % | 91.6 % | 31.3 % | 95.2 % | 54 % |
| CS-2 | 54.7% | 31.1% | 25.6% | 0.4 % | 86.9 % | 76.6 % | 96.4 % | 69.1 % |
| CS-3 | 48.5% | 19.2% | 28.0% | 9.0 % | 95.7 % | 71.9 % | 98.4 % | 64.6 % |
| CS-4 | 21.4% | 16.8% | 12.6% | 0.8 % | 100 % | 74.6 % | 100 % | 66.4 % |
| CS-5 | 0 % | 0 % | 0 % | 1.1 % | 89.6 % | 54.9 % | 100 % | 67.7 % |
| CS-6 | 24.8% | 11.5% | 15.5% | 6.7 % | 95.6 % | 88.8 % | 106 % | 67.0 % |
| CS-7 | 13.2% | 7 % | 0 % | 1.5 % | 91.6 % | 67.7 % | 97.0 % | 54 % |
| CS-8 | 12.7% | 2 % | 1.4 % | 0.7 % | 81.8 % | 61.8 % | 79.5 % | 49.4 % |
| CS-9 | 0 % | 0 % | 1.5 % | 0.2 % | 73.8 % | 46.9 % | 83.9 % | 77 % |
| CS-10 | 4.8% | 0.2% | 7.2% | 1.1 % | 68.9% | 68.2 % | 83.6 % | 52.3 % |
| CS-11 | 76.5% | 40.0% | 35.1% | 10.9 % | 140 % | 59.4 % | 100 % | 50.7 % |
| CS-12 | 0.3 % | 0 % | 0.35 % | 0 % | 110% | 74.2 % | 98.6 % | 91.1 % |
| CS-13 | 60.4% | 24.0% | 66.7% | 11.6 % | 74.0 % | 65.8 % | 93.5 % | 87.4 % |
| CS-14 | 63.3% | 35.8% | 33.1% | 17.5% | 74.2 % | 71.6% | 100 % | 51.9 % |
| CS-15 | 5.3% | 0% | 3.4% | 0% | 95.7 % | 71.9 % | 98.4 % | 64.6 % |
| CS-16 | 46.8% | 9.3% | 28.8% | 1.2% | 87.9% | 20 % | 95.9 % | 10.9 % |
| CS-17 | 51.4% | 15.8% | 47.3% | 8.9% | 102.0 % | 48.3 % | 108.2 % | 92.3 % |
| CS-18 | 49.9% | 8.3% | 7.1% | 0.8% | 114.8 % | 92.2 % | 68.2% | 71.5 % |
| CS-19 | 63.5% | 21.1% | 35.4% | 12.8% | 96.9 % | 87.9 % | 93.0 % | 101 % |
| CS-20 | 65.5% | 24.1% | 38.4% | 15.8% | 103.9 % | 88.9 % | 91.0 % | 100 % |

"a" represents the bacterial viability after the pHly-1 NPs and CHX treatment for 10 min. The killing activity of pHly-1 NPs and CHX was measured against clinically isolated *S.mutans* strains by counting the CFU of alive bacteria. Bacterial survival

(%)= CFU of bacteria treated with pHly-1 NPs or CHX/CFU of bacteria treated with ultrapure water×100%.

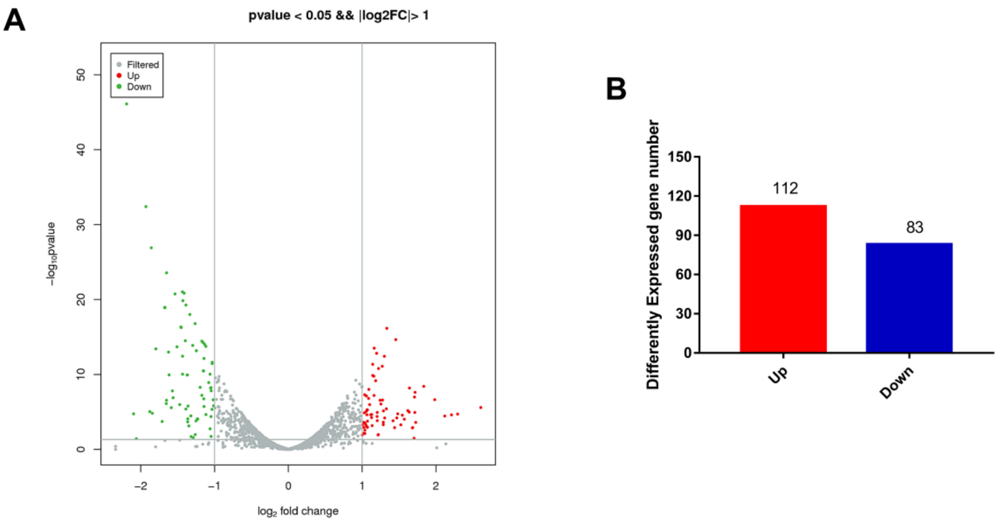


**Supplementary Fig.6. A** and **B**, Volcano map (**A**) and statistical histogram (**B**) of differentially expressed genes (DEGs identified between *S.mutans* treated with 22 μM pHly-1 NPs for 1 h and with control.

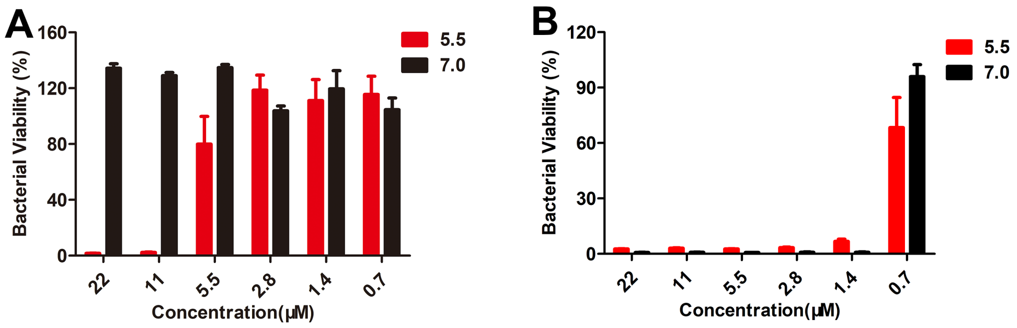


**Supplementary Fig.7. A and B, The determination of $MBIC_{50}$ of pHly-1 NPs (A) and CHX (B) at pH 5.5 and 7.0, respectively.** The MTT assay was performed to determined the minimum biofilm inhibition concentration ($MBIC_{50}$) by using the Microplate Reader at OD 490 nm. The bacterial viability(%)=$[(A490_{sample}-A490_{blank})/(A490_{negative\ control}-A490_{blank})]\times 100\%$ All of the data are presented as average± SD. Each experiment consists of 3 repetitions.

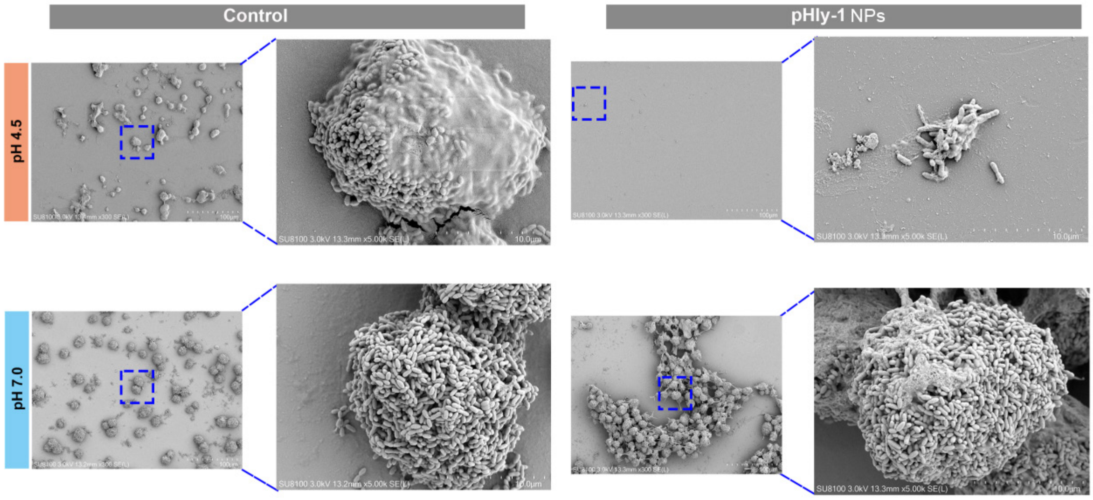


**Supplementary Fig.8. The effect of pHly-1 NPs on the initial adhesion of *S.mutans* UA159 at different pHs.** The experimental procedure was similar to that to the $MBIC_{50}$ determination except for the addition of bacteria to glass slides in 24 well plates. The treatment concentration of pHly-1 NPs was 11 μM and after washing with PBS the adherent bacteria on the glass slide surface were characterized by SEM.

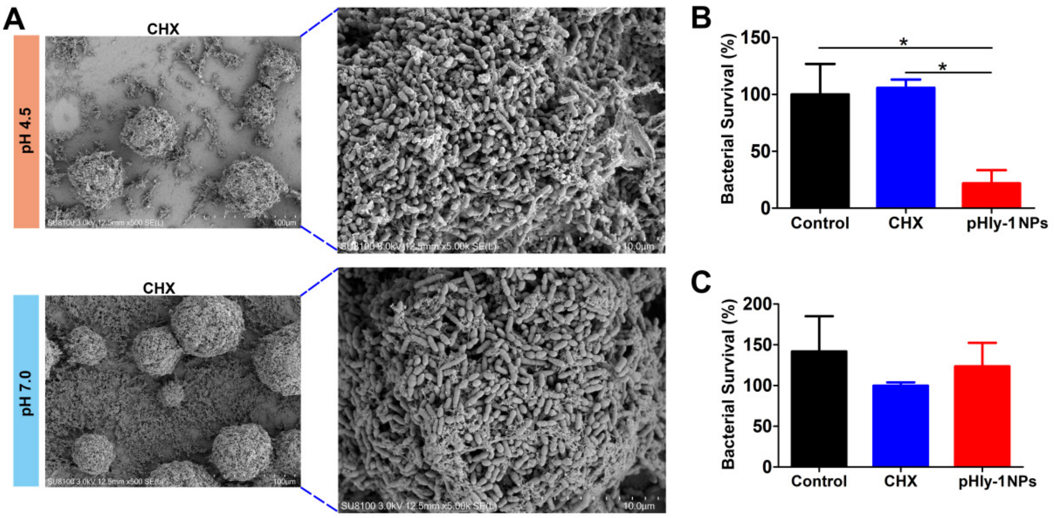


**Supplementary Fig.9. A, The effect of CHX on *S.mutans* UA159 biofilm development at different pHs as determined by scanned electronical microscopy (SEM).** The red arrows and blue arrows represent EPS and *S.mutans* UA159, respectively. **B** and **C**, Quantitative analysis of the total viable cell count after the biofilms on glass slides were treated with control, CHX and pHly-1 NPs, respectively at pH 4.5(**B**) and 7.0 (**C**). The biofilm was removed and homogenized by sonication. The biofilm suspension was diluted, plated, and incubated for 48 h at 37 °C. All of the data are presented as average± SD. Each experiment consists of 3 repetitions.

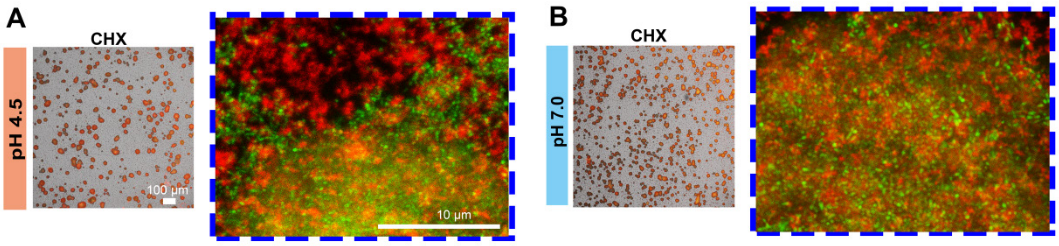


**Supplementary Fig.10 A** and **B**, The effect of CHX on *S.mutans* UA159 biofilms development at pH 4.5 (**A**) and pH 7.0 (**B**) as determined by Laser scanning confocal microscopy (LSCM). The red fluorescence represented the EPS and green fluorescence represented *S.mutans*.

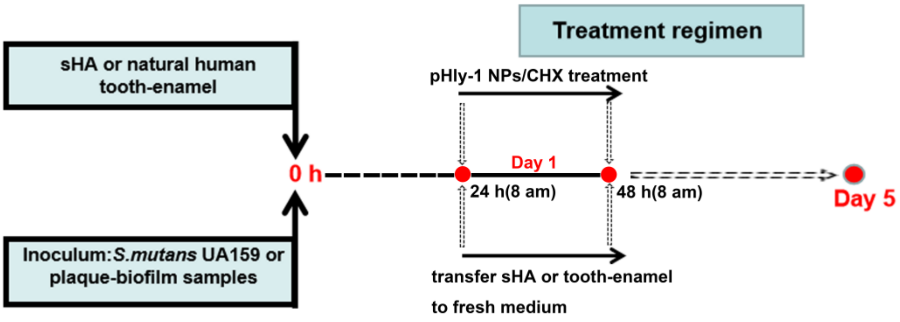


**Supplementary Fig.11. Treatment regimen of *Ex vivo* biofilms.**

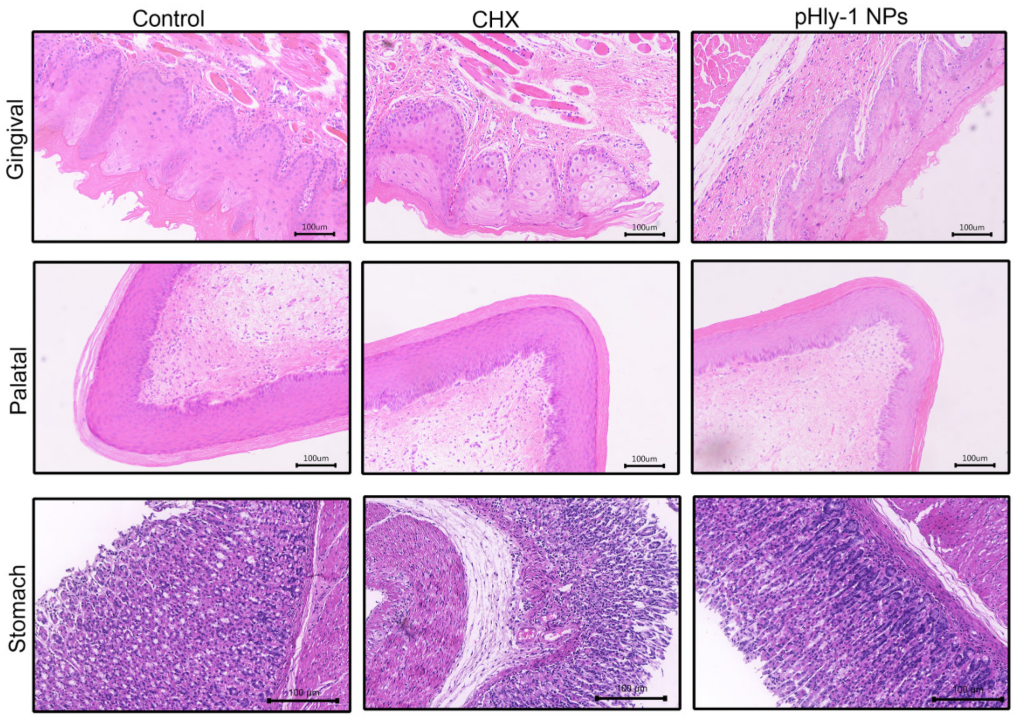


**Figure S12. H&E staining of gingival, palatal and gastric tissues** (Scale bar: 100 μm)

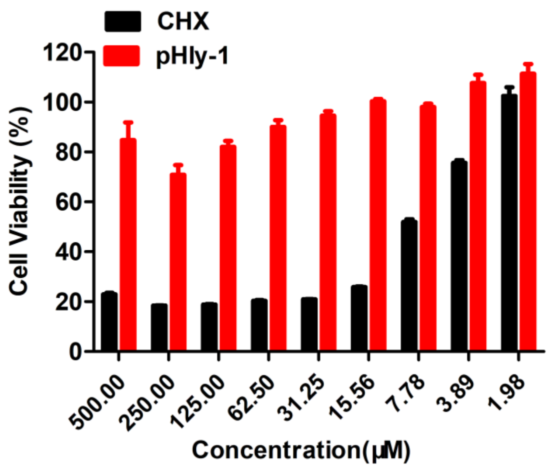


**Supplementary Fig.13. The toxicity of pHly-1 NPs to human gingival fibroblast (HGF-1) was assessment by CCK-8 assay.** DMEM medium(10 % FBS) was used for cell culture and DMEM medium was used for drug preparation in toxicity tests. The All of the data are represented as average±SD. Each experiment consists of 3 repetitions.

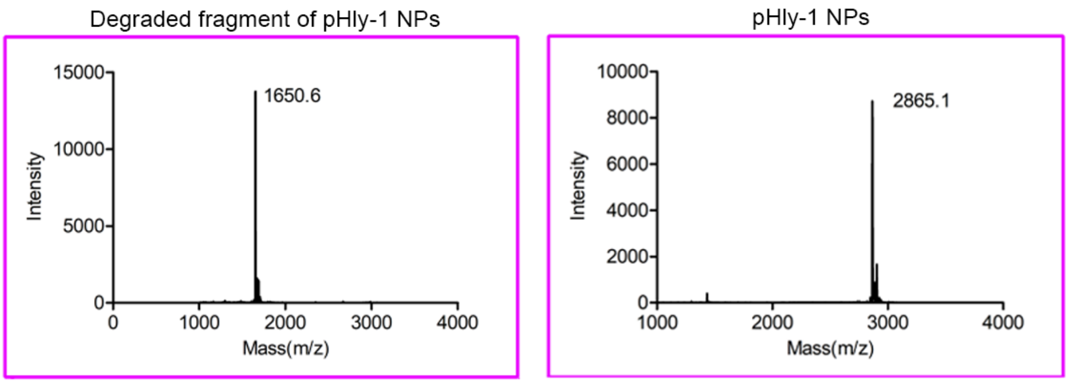


**Supplementary Fig.14. MALDI-TOF MS analysis of pHly-1 NPs degradation by pepsin.**

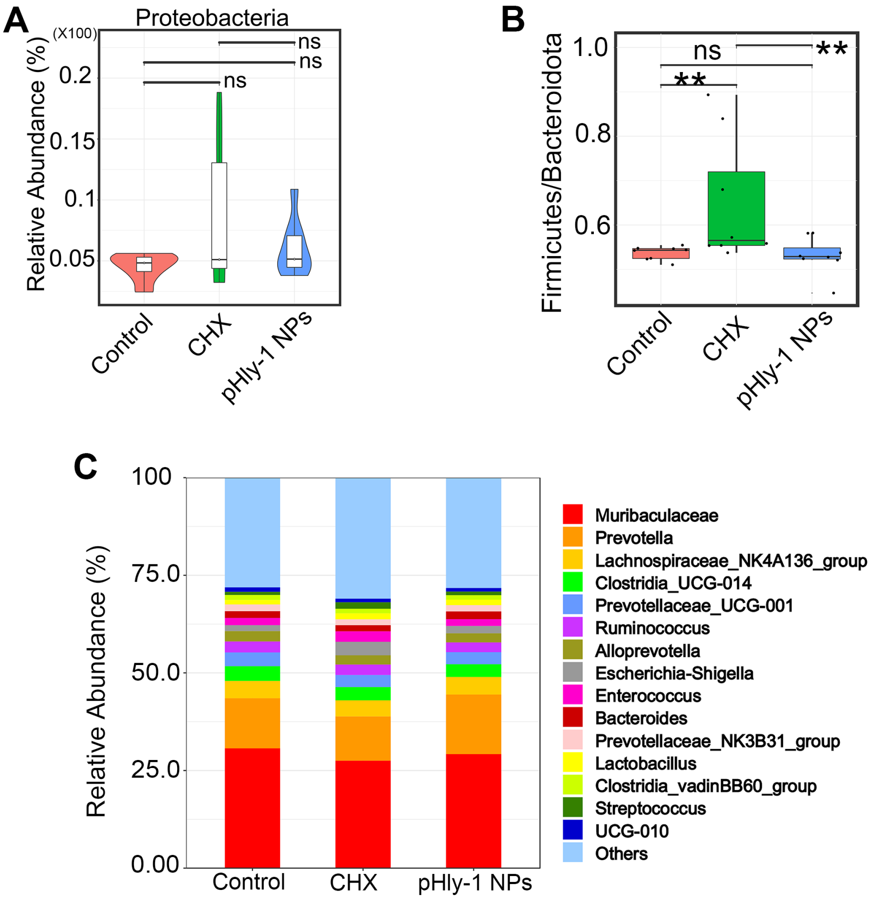


**Supplementary Fig.15. Analysis of phyla and genus of bacteria following pHly-1 NPs or CHX treatment. A**, Relative abundance of the bacterial phyla(Proteobacteria). **B**, The ratio between the two main phyla (Firmicutes: Bacteroidetes) following the pHly-1 NPs or CHX treatment. Wilcoxon test was used for statistical analysis. Data are shown as mean±s.d.(n=8).C, Relative abundance of the top 30 bacterial genus.
