# Supplementary material for: Dual-sensitive antibacterial peptide nanoparticles prevent dental caries
Source: Theranostics. 2022 Jun 13;12(10):4818–33. doi: 10.7150/thno.73181 (PMC9254247; doi:10.7150/thno.73181)
Supplement: Supplementary file 2 — Supplementary methods. [file thnov12p4818s2.pdf]

Methods

Peptide and different pH of culture medium prepared. The pH of BHI medium was adjusted by HCl and NaOH. All peptides, including lycosin-I, pHly-1, s-pHly-1 were synthesized on a Rink amide-AM resin using the SPPS (9-fluorenyl-methoxycarbonyl solid-phase peptide synthesis) method as reported previously[1]. Then peptides were cleaved from the resin using TFA/1,2-ethanedithiol/thioanisole/anisole (90/5/3/2 (v/v)), precipitated in cold diethyl ether and the collected precipitate was dried by air-drying. The prepared peptides were purified by reverse phase-HPLC (RP-HPLC) and their molecular weights were determined by MALDI-TOF MS.

Hemolytic assay. Approximately 4 % (in volume) of the mouse erythrocytes were prepared by diluting fresh erythrocytes to 25-fold dilution with PBS buffer (pH 7.4). The stock peptide solution (10 M) diluted by PBS solution to reach various concentrations (200 μL) was placed in a 1.5 mL sterile microfuge tube, followed by the addition of 200 μL of erythrocyte suspension. The mixture was incubated at 37 °C for 30 min. Then, the mixture was centrifuged at 3,500 rpm for 5 min to collect the supernatant. Aliquots (100 μL) of the supernatant were removed to 96-well plates, and the absorbance was detected at 450 nm using a microplate reader (Multiskan Spectrum, BioTek Inc., USA). Untreated erythrocyte suspension in an equal volume of PBS solution and 0.1% Triton X-100 were used as the negative control and positive control, respectively.

Isolation and culture of clinical *S.mutans* strains. Ethical approval of the study and the written consent/permission forms were obtained from Institutional Review Board (IRB) at Hunan Normal University prior to the study. For each child, written permission form was reviewed and signed by their legal guardians. Plaque-biofilm samples were collected from patients, including 16 Middle-aged and young patients and 4 children (age between 48 and 72 months) diagnosed with severe early childhood caries (S-ECC) in Changsha Stomatological Hospital. After being collected using a sterilized periodontal scaler, pooled plaque samples were transferred into 1 mL thiolglycollate solution, covered with 0.5 mL sterile paraffin wax and immediately transported to the laboratory for isolation. In order to sufficient disperse the aggregates, samples should be gently vortexed and sonicated before isolation. A series of diluted dispersed samples were plated on TYCSB at 37 ^{o}C under anaerobic conditions. Then, the suspicious clones were selected and plated on the mitis salivarius agar (MSA) plus bacitracin at 37 ^{o}C under anaerobic conditions for further isolation. The isolated *S.mutans* were most of identified by mass spectrometry and all identified by microbiochemical reaction again. All isolated *S.mutans* were stored with glycerol for subsequent experiments.

Minimal inhibition concentration (MIC) determination. *S.mutans* UA159 (ATCC 700610) and 20 clinical *S.mutans* strains were used. *S.mutans* UA159 was purchased from Shanghai Biofeng Biotechnology Co., LTD. These bacteria grown in brain heart infusion broth (BHI, Beijing Land Bridge, China) at 37 °C under anaerobic conditions. Due to bacteria incubation under the condition of excessive acid for a long time will affect its vitality, therefore, pH 5.5 was choose as acidic condition for the MIC determination. Peptides and CHX were dissolved in BHI medium of different pHs (pH 5.5 and 7.0) using serial dilutions to obtain different diluted solutions. 2 μL of bacteria (1×10^7 CFU (colony forming units) in the logarithmic phase were added into each well of 96-well plates containing 200 μL different diluted solutions. Note that peptides also showed absorption value when detected with a microplate reader due to the aggregate behavior of peptides. In order to avoid interfering with the MIC test, it is important to set up the control wells which only contain different concentrations of peptides in BHI medium of different pHs. The absorbance at 600 nm was detected with a microplate reader (Multiskan Spectrum, BioTek Inc., USA) after 48 h. The inhibition rate of drugs on bacteria was calculated based on the absorbance OD_{600}(A600). The bacterial viability(%)=[($A600_{sample}$-$A600_{blank}$)/($A600_{negative\ control}$-$A600_{blank}$)] ×100%.

Bacterial killing by pHly-1 NPs at various pHs. pHly-1 NPs were diluted in BHI medium of different pHs (pH 4.5, 5.5, 6.8, and 7.0) to obtain specific diluted solutions (final volume of 200 μL). 1 μL bacterial suspension (2×10^8 CFU/mL) in the logarithmic phase was added into corresponding diluted solutions and mixed individually. After being incubated for 10 min at 37 °C under anaerobic conditions, aliquots of 20 μL bacterial suspension were plated on BHI agar plates. The bacterial killing activity of pHly-1 NPs was measured by counting the CFU of alive bacteria with agar plating after incubation under anaerobic conditions for 48 h at 37 °C. The results were determined and presented of three repeats. Bacterial survival(%) = CFU of bacteria treated with drugs/CFU of bacteria treated with negative control×100% [2].

The SYTO 9 / PI double staining and scanning electron microscopy (SEM) were also performed to evaluate the bacterial killing and bacterial membrane disruption. For SYTO 9/PI double staining, pHly-1 NPs was added to actively growing *S. mutans* UA159 (10^6 CFU/mL) at the concentration of 22 μM at pH 4.5 or pH 7.0, after 1 h incubation, the bacteria treated were imaged by using LIVE/DEAD BacLight Bacterial Viability Kit (Thermo Fisher Scientific). SYTO 9 and PI were added according to the manufacturer's protocol and used for labeling bacteria. After washing and centrifuging, the bacterial suspension was dropped on a glass slide covered with a slip and observed under a fluorescence microscope.

For SEM, *S. mutans* UA159 was treated by the same way to SYTO 9/PI double staining asay and then harvested, washed, and then fixed in 2.5 % glutaraldehyde for 3 h. After dehydration by serial concentrations of ethanol solutions (10%, 25%, 50%, 75%, 95%, and 100%) and lyophilization overnight, the lyophilized samples were coated with gold and then photographed by SEM.

Characterization analysis of pHly-1 NPs at different pHs. The morphology of pHly-1 NPs at pH 4.5 and 7.0 were analyzed by TEM and AFM, respectively [3,4]. TEM images were taken by a Tecnai G2 F20 microscope with an accelerating voltage of 100 kV. The final concentration of TEM samples of pHly-1 NPs was 100 μM. An 8 μL TEM sample was pipetted onto the carbon-coated copper grid surface for 3 min and removed by filter paper. Then, 8 μL of 2 wt % uranyl acetate was dripped onto the grid for staining 3 min. After removing the droplet with filter paper, the grid was dried in a desiccator before measurement.

For AFM characterization, 8 μL of the peptide samples, at the concentration of 100 μM at pH 4.5 or 7.0, were dropped to the freshly cleaved mica surfaces, sit for 5 min and the retained solution was removed by filter paper. After the samples were dried under atmospheric conditions, the images were taken by BenYuan instrument under the tapping mode.

The secondary structure of pHly-1 NPs in different solutions at pH 4.5 and 7.0 was determined by Jasco-810 spectropolarimeter using 2 mm quartz slides at 25 °C. According to the method reported in the literature[5] to prepare liposome required in the secondary structure test. Briefly, lipids were dried from chloroform under vacuum overnight.The vesicles thus formed were then extruded 11 times through a 0.1 μm nucleopore polycarbonate filter (two filters, 100 nm pore size) using a Avanti extruder system to give large unilamellar vesicles of a 100-nm diameter. All spectra were recorded with a wavelength interval of 0.5 nm. The final concentration of peptide samples was 100 μM. In addition, Zeta (ζ) potentials and hydrodynamic sizes of pHly-1 NPs were measured using a Zetasizer (Malvern Instruments, UK). The measurements were performed in the concentration of 100 μM at pH 4.5 and 7.0, respectively.

RNA-seq Analysis. *S.mutants* UA159 in the logarithmic growth phase were treated with 22 μM pHly-1 NPs in 1 mL BHI medium (pH 4.5) for 1 h. Total bacterial RNA was extracted by using a mirVana miRNA isolation Kit (ambion). The quality and quantity of the RNA were assessed by NanoDrop 2000/2000c (Thermo Scientific, Wilmington, DE, USA). RNA integrity number (RIN) was analyzed by Agilent 2100 Bioanalyzer (Agilent Technologies, Santa Clara, CA, USA). RiboZero rRNA removal kit (Epicentre, USA) for gram-positive was used to eliminate ribosomal RNA before RNA sequencing analysis. Strand-specific cDNA libraries were generated by standard procedures for ensuing Illumina sequencing using the Illumina Nova 6000 platform (Shanghai Oebiotech Co., Ltd., Shanghai, China) and 150 bp double-ended readings were generated. Raw data sequencing was processed by Trimmomatic. The cleaned reads were mapped to the *S.mutans* reference genome using Rockhooper2[6]. The FPKM value of each transcript was calculated with Rockhooper2. Differentially expressed transcripts were identified by DESeq R package.

Minimum biofilm inhibition concentration ($MBIC_{50}$) determination. The $MBIC_{50}$ determination was adapted from the reported literature[7]. *S.mutans* UA159 in the logarithmic phase was diluted in BHI (pH 5.5 or 7.0) containing 1 % sucrose to obtain

a concentration of 1×10^8 CFU/mL. pHly-1 NPs and CHX were diluted in BHI containing 1% sucrose to reach a series of concentrations and added to 96-well microplates (180 μL). Then, 2 μL *S.mutans* suspensions were seeded into 96-well microplates in the presence of different concentrations of pHly-1 NPs and CHX and the plate was incubated at 37 °C. After 24 h, the culture supernatants were gently discarded, and each well was rinsed three times with PBS (pH 7.0) to remove unattached bacteria. The bacterial viability was measured by methyl thiazol tetrazolium (MTT) assay. The lowest concentration of pHly-1 NPs or CHX that inhibited biofilm by 50% compared to the untreated culture control was the minimum biofilm inhibitory concentration ($MBIC_{50}$). The MTT assay was performed to determined the minimum biofilm inhibition concentration ($MBIC_{50}$) by using the Microplate Reader at OD 490 nm [7]. The bacterial viability (%)=[($A490_{sample}$-$A490_{blank}$)/($A490_{negative\ control}$-$A490_{blank}$)]×100%.

***S.mutans* biofilm treatment *in vitro*, characterization and quantitative analysis.** Biofilms were formed on circular glass slides in a 24-well plate. Specifically, *S. mutans* UA159 in the logarithmic growth phase was adjusted to a final density of 2×10^6 CFU/mL with BHI medium supplemented with 1% sucrose. 12 h after biofilm formation, the circular glass slides were removed and placed in another new 24-well plate for treatment. Before treatment, pHly-1 NPs and CHX were diluted with pH 7.0 or 4.5 PBS to obtain a final concentration of 55 μM. The glass slides were washed three times (1 min/time) with PBS (pH 4.5 or 7.0), and then 55 μM pHly-1 NPs or CHX was added into corresponding wells. After 10 min treatment, the glass slides were washed three times with pH 7.0 or 4.5 PBS and then transferred into a new 24-well plate containing fresh BHI medium supplemented with 1 % sucrose. This treatment regimen was administered every 12 h for a total of 3 times. Biofilms formed on glass slides were used for SEM, fluorescent staining and bacterial quantitative analyses. For SEM analysis, biofilms were fixed with glutaraldehyde and the characterization method was similar to the planktonic bacteria described above.

For fluorescent staining[9], after the treatment was finished, 1 μM Alexa Fluor 647-labeled dextran conjugate (Thermo Fisher Scientific) was added to the BHI medium to label the EPS. 3 min later, the bacteria in biofilms were labeled with 2.5 μM SYTO 9 (Thermo Fisher Scientific). Biofilms on the glass slides were washed 3 times and then observed by using laser scanning confocal microscopy (LSCM) (Leica SP8, Germany).

For quantitative analysis[9,10], after the treatment was finished, biofilms were removed from the glass slides and homogenized by water bath sonication followed by probe sonication at an output of 7 W for 30 s (Branson Sonifier 150, Branson Ultrasonics). After biofilm homogenates were diluted, 20 μL suspension was plated on BHI agar plates. The effect of pHly-1 NPs or CHX on biofilms was measured by counting the CFU of alive bacteria with agar plating after incubation for 48 h at 37 °C. Bacterial survival(%) = CFU of bacteria treated with drugs/CFU of bacteria treated with negative control×100%.The results were determined and presented of three repeats.

Treatment and quantitative analysis of biofilm model on sHA. *S.mutans* biofilms also formed on sHA blocks (surface area=1 ± 0.2 cm^2) in 24-well plates. According to the literature[10], before the experiment, hydroxyapatite (diameter, 1.25 cm; Kunshan Chinese technology new materials CO, LTD) was coated with filter-sterilized clarified human whole saliva. The biofilm formation, treatment regimen, and quantitative analysis were roughly similar to the biofilms formed on circular glass slides as described above. The differences are that the biofilms should be treated after 24 hours of formation and biofilms were treated every other day for a total of 5 days. In addition, after treatment for 5 days, the sHA blocks were washed with PBS (pH 7.0) and then fixed with 2.5 % glutaraldehyde for 10 min. After being washed another 3 times with PBS (pH 7.0) to remove the remaining glutaraldehyde, the sHA blocks were stained using 0.5% crystal violet for 20 min. The fully washed sHA blocks were observed under a light microscope (Insein Li Fung1080P 60FPS, China).

Treatment and quantitative analysis of human derived *ex vivo* biofilms. Ethical approval of the study and the written consent/permission forms were obtained from Institutional Review Board (IRB) at University of Hunan Normal University prior to the study. For each patient, written permission form was reviewed and signed. Human derived *ex vivo* biofilm model was performed to further assess the antibiofilm efficacy of pHly-1 NPs (referenced and adapted from Y Liu et al[10]).The plaque-biofilm samples of 4 children were divided into two parts: one is used for isolation of *S. mutans* as described above, and the other was preserved with glycerol. The total cultivable bacteria for *ex vivo* biofilm formation was measured to ensure the same density of added bacteria (~2×10^8). To ensure the similar *S. mutans* proportion for inoculum to the greatest extent, the total cultivable bacteria was consisted of 90 % *S. mutans* which was isolated and amplified from the plaque-biofilm samples and 10 % preserved homogenized pooled plaque sample. The biofilms formed on sterilized human enamel blocks in 24-well plates and the blocks were inoculated with cultivable bacteria in BHI containing 1 % sucrose at 37 °C and 5 % CO_2. The treatment regimen was similar to the biofilms formed on sHA blocks as described above. After 5 days, enamel blocks were fixed with 2.5 % glutaraldehyde for 10 min and washed three times with PBS (pH 7.0). Optical 3D surface profilometer (SuperView W1, CHOTEST) was used to analyze the three-dimensional morphology. The quantitative analysis was measured by counting the CFU of alive bacteria with agar plating after incubation for 48 h at 37 °C. Bacterial survival (%) = CFU of bacteria treated with drugs/CFU of bacteria treated with negative control×100%.

***In vivo* rodent model simulating severe childhood caries.** All the animal studies were approved by the Institutional Animal Care and Use Committee (IACUC) of Hunan Normal University, and the National Institutes of Health guidelines for the performance of animal experiments were followed. 15 days-old female Sprague–Dawley rat pups were purchased with their dams from Slac & Jingda Corporation of laboratory animals, Changsha, China. All animals should be checked for the pathogen

infections especially for the *S.mutans* by plating oral swabs on mitis salivarius agar plus bacitracin. The animals were then infected by mouth with *S. mutans* UA159 (mid-logarithmic) 4 times (once a day) by pipetting 0.1 mL of a cell suspension containing 2×10^8 colony-forming units into their mouths. When the infection was confirmed, all the pups were randomly placed into three treatment groups, and their teeth were treated topically once a day for a total 35 days using the swabs plus modified syringe. The concentration of CHX and pHly-1 NPs was 100 μM and the volume was 300 μL. The three treatment groups were: (1) control (PBS, pH 4.5); (2) CHX (100 μM, dissolve in PBS, pH 4.5); (3) pHly-1 NPs (100 μM, dissolve in PBS, pH 4.5). Each group was provided the National Institutes of Health cariogenic diet 2000 and water containing 5% sucrose. After 35 days of treatment, all animals were sacrificed and jaws were collected according the literature. Determination of caries score of the jaws was performed by a calibrated examiner who was blind for the study by using codified samples according to Larson's modification of Keyes' system [11-13].

Toxicity tests on human organoids. Organoids have been established in our lab. Ethical approval of the study and the written consent/permission forms were obtained from Institutional Review Board (IRB) at Hunan Normal University before the study commencement. For each patient, written permission form was reviewed and signed by themselves. According to the literature[14,15], briefly, the tissue samples obtained from patients were removed excess fat or muscle tissues and cut into small fragments. The fragments were washed thoroughly, then digested and centrifuged to obtain cell pellet. The resulting pellet was resuspended in ice-cold 70 % BME for incubating 15 min and then the organoid medium was added. The composition of oral and gastric organoid medium was as follows:

Human oral organoid medium contains Advanced Dulbecco's Modified Eagle's Medium/F12 supplemented with penicillin/streptomycin, 10 mM HEPES, 2 mM GlutaMAX, 1×B27 supplement (Life Technologies), 1.25 mM N-acetyl-l-cysteine (Sigma-Aldrich), 10 mM Nicotinamide (Sigma-Aldrich), 50 ng/mL human EGF (PeproTech), 500 nM A83-01(Tocris Bioscience), 10 ng/mL human FGF10 (PeproTech), 5 ng/mL human FGF2 (PeproTech), 1 μM Prostaglandin E2 (Tocris Bioscience), 3 μM CHIR 99021 (Sigma-Aldrich), 1μM Foskolin, 100 μg/mL Primcoin ,10 μM Y-27632 (Wako).

Human gastric organoid medium contains Advanced Dulbecco's Modified Eagle's Medium/F12 supplemented with penicillin/streptomycin, 10 mM HEPES, 2 mM GlutaMAX,1×B27 supplement (Life Technologies), 50 ng/mL human EGF (PeproTech), 200 ng/mL human FGF10 (PeproTech), 1.25 mM N-acetyl-l-cysteine (Sigma-Aldrich), 10 nM Gastin-l, 500 nM A83-01 (Tocris Bioscience), 10 μM Y-27632 (Wako), 100 μg/mL Primcoin, 5 μM SB202190 (Sigma-Aldrich), 1 μM Prostaglandin E2 (Tocris Bioscience), 100 ng/mL human recombinant IGF-1 (Bio Legend), 3 μM CHIR 99021 (Sigma-Aldrich), 10 mM Nicotinamide (Sigma-Aldrich).

For the organoid toxicity assay, organoids were digested into single cells. After centrifugation and washing, single cells were plated in 70 % BME and then added to 96-well plates (5 μL/well). After that, 100 μL organoid medium was added into the

wells and changed every 3 days for a total of 8 days. At the 9th day, pHly-1 NPs and CHX were diluted by the organoid medium to obtain a serial of concentration solutions and these solutions were added into the corresponding well. After incubation for 1 days, the organoid viability was measured using CellTiter-Glo 3D cell viability assay.

Toxicity tests on mice. Furthermore, 35-40 g female Kuming mice were also purchased from Slac & Jingda Corporation of laboratory animals, Changsha, China to systematically evaluate the toxicity of pHly-1 NPs to oral microflora and soft tissue in mice. All animals were randomly placed into three treatment groups, and pHly-1 NPs or CHX in aqueous solution was gradually added to mice mouth once a day for seven days. Considering the concentration of CHX used in clinic is usually 0.2 %, we determined that the concentration of the pHly-1 NPs rinsed mouse's mouth is also 0.2 % and the volume was 100 μL. At the 8th day, saliva was drawn for oral microbiome analysis, and gingival, palatal and gastric tissues were collected for histopathological analysis.

Oral flora 16S rRNA sequencing. Total genomic DNA of oral samples was extracted in OE Biotech Company (Shanghai, China) using a QIAamp DNA Stool Mini Kit (Qiagen, Germantown, MD, USA). Bacterial DNA was amplified with primers targeting the V3-V4 regions (50-TACGGRAGGCAGCAG-30, 50-AGGGTATCTAATCCT-30) and the final amplicon was quantified using a Qubit dsDNA assay kit (Life Technologies, California, United States). Equal amounts of purified amplicons were pooled for subsequent sequencing by the Illumina Nova 6000 platform (Illumina, USA) (Shanghai Oebiotech Co., Ltd., Shanghai, China). The raw data were treated and processed using the QIIME software package (version 1.8.0). The operational taxonomic units (OTUs) were generated using Vsearch software with a 97% similarity cutoff. The QIIME package and the RDP classifier (confidence threshold was 70%) were performed to select the representative read of each OTU and annotate and BLAST against the Silva database Version 123, respectively. Furthermore, the microbial diversity in oral samples was estimated by using the alpha diversity that include Chao1 and Shannon index. The bacterial diversity between groups was compared using the Wilcoxon rank sum test.

References

1. Zhang, P., *et al.* Position Effect of Fatty Acid Modification on the Cytotoxicity and Antimetastasis Potential of the Cytotoxic Peptide Lycosin-I. *J Med Chem* **62**, 11108-11118 (2019).
2. Xiong M, Bao Y, Xu X, Wang H, Han Z, Wang Z, Liu Y, Huang S, Song Z, Chen J, Peek RM Jr, Yin L, Chen LF, Cheng J. Selective killing of Helicobacter pylori with pH-responsive helix-coil conformation transitionable antimicrobial polypeptides. *Proc Natl Acad Sci U S A* **114**, 12675-12680(2017).
3. Hu, B., *et al.* Noncanonical Amino Acids for Hypoxia-Responsive Peptide Self-Assembly and Fluorescence. *Journal of the American Chemical Society* **143**, 13854-13864 (2021).
4. Li M, Song Y, Song N, Wu G, Zhou H, Long J, Zhang P, Shi L, Yu Z. Supramolecular Antagonists Promote Mitochondrial Dysfunction. Nano Lett, **21**,5730-5737(2021).
5. Akishiba M, Takeuchi T, Kawaguchi Y, Sakamoto K, Yu HH, Nakase I, Takatani-Nakase T, Madani F, Gräslund A, Futaki S. Cytosolic antibody delivery by lipid-sensitive endosomolytic peptide. *Nat Chem*. 2017;9(8):751-761.
6. Tjaden, B. De novo assembly of bacterial transcriptomes from RNA-seq data. *Genome Biol* **16**, 1 (2015).
7. Bekhouche, M., *et al.* Development of an antibacterial nanocomposite hydrogel for human dental pulp engineering. *J Mater Chem B* **8**, 8422-8432 (2020).
8. Zhong H, Xie Z, Wei H, Zhang S, Song Y, Wang M, Zhang Y. Antibacterial and Antibiofilm Activity of Temporin-GHc and Temporin-GHd Against Cariogenic Bacteria, Streptococcus mutans. *Front Microbiol* **10**, 2854(2019).
9. Xiao, J., *et al.* The exopolysaccharide matrix modulates the interaction between 3D architecture and virulence of a mixed-species oral biofilm. *PLoS Pathog* **8**, e1002623 (2012).
10. Liu, Y., *et al.* Topical ferumoxytol nanoparticles disrupt biofilms and prevent tooth decay in vivo via intrinsic catalytic activity. *Nat Commun* **9**, 2920 (2018).
11. Bowen, W.H. Rodent model in caries research. *Odontology* **101**, 9-14 (2013).
12. Keyes, P.H. Dental caries in the molar teeth of rats. I. Distribution of lesions induced by high-carbohydrate low-fat diets. *J Dent Res* **37**, 1077-1087 (1958).
13. Wang, Y., *et al.* Antimicrobial peptide GH12 suppresses cariogenic virulence factors of Streptococcus mutans. *J Oral Microbiol* **10**, 1442089 (2018).
14. Driehuis, E., *et al.* Oral Mucosal Organoids as a Potential Platform for Personalized Cancer Therapy. *Cancer Discov* **9**, 852-871 (2019).
15. McCracken, K.W., *et al.* Modelling human development and disease in pluripotent stem-cell-derived gastric organoids. *Nature* **516**, 400-404 (2014).
